# Contemplative practices serve as complementary mental health strategies in nationally representative samples from Australia and New Zealand

**DOI:** 10.1038/s41598-026-51375-4

**Published:** 2026-05-19

**Authors:** Karin Matko, Cate Bailey, Julieta Galante, Jonathan N. Davies, Nicholas T. Van Dam

**Affiliations:** 1 Contemplative Studies Centre, Melbourne School of Psychological Sciences, University of Melbourne, Melbourne, Australia; 2 Melbourne Health Economics, School of Population and Global Health, University of Melbourne, Melbourne, Australia

**Keywords:** Contemplative practices, Meditation, Yoga, Breathing techniques, Relaxation, Mental health, Complementary and alternative medicine, Representative survey, Health care, Psychology, Psychology

## Abstract

Contemplative practices (including meditation and yoga) are increasingly popular worldwide, especially as alternatives and/or complements to mental health treatment. However, nationally representative samples that allow for accurate estimates of prevalence and relation to mental health are scarce. We conducted a nationally representative, cross-sectional survey in Australia and New Zealand (*N* = 2640). Sampling was stratified by age, gender, ancestry/ethnicity, region and income. Two pre-registered questions were analysed to explore participants’ engagement in contemplative practices and associations to psychological distress and mental healthcare use. We used posthoc regression analyses to further clarify relationships between variables. Overall, 70% of participants engaged in contemplative practices over the past year, most commonly meditation (31%), relaxation (25%), breathing techniques (24%), and yoga (21%). Improving health and wellbeing were the primary motivations for practice. All contemplative practice users reported significantly higher psychological distress and mental healthcare use than non-practitioners (*p*’s<0.05). Adjusting for sociodemographic differences removed associations with distress among yoga and relaxation practitioners (β_relaxation_ = 0.02, β_yoga_ = − 0.03, *n.s.*). For meditators, the association disappeared when we accounted for mental healthcare use (β_meditation_ = 0.01, *n.s.*). Breathing practice was associated with increased distress in all models (β_breathing_ = 0.04–0.11, *p’s<*0.05). Notably, meditators and relaxation practitioners with unmet healthcare needs reported less distress than non-practitioners with unmet needs (*d*_*meditation*_=0.38, *d*_*relaxation*_=0.35). Contemplative practices are widespread among Australians and New Zealanders and may play a complementary and/or alternative role in managing mental health. Further research is needed to study their complex associations with mental health, and which types of contemplative practices are beneficial and safe for whom.

**Supplementary Information:**

The online version contains supplementary material available at 10.1038/s41598-026-51375-4.

## Introduction

Given that approximately one third of the world’s population is likely to develop a mental disorder in their lifetime^1^, there is pressing need for cost-effective mental health interventions. Evidence suggests a 24% global increase in psychological distress from 2009 to 2021^2^. Globally, there are extremely high levels of unmet mental healthcare need; among those with depression in high-income countries, only 51% have treatment coverage while only 23% have minimally adequate coverage^3^. Access to care is even more limited in low and middle-income countries^4^. As offerings that typically make use of efficient group-based delivery^5–7^, contemplative practices like meditation and yoga, when effective, could help address this service gap. In an analysis of nationally representative data in the US, we previously identified an association between contemplative practice use and unmet (mental) health needs^8^.

Contemplative practices are defined as forms of mental training that emphasize self-awareness, self-regulation, and/or self-inquiry^9^ and encompass a wide variety of practices, such as meditation, yoga and qigong. Some contemplative practices have been studied intensively in the past decades as means to improve physical and mental wellbeing. In a recent meta-analysis, mindfulness-based programmes (structured meditation training) demonstrated robust salutary effects^10^, a finding that generally holds across a wide range of study populations and clinical conditions, particularly those related to mental health^11^. Likewise, in a review of meta-analyses, yoga interventions were found to be beneficial for a wide range of physical and psychological conditions^12^. Studies conducted in Australia, Germany, UK, and the US suggest that practitioners of contemplative practices often utilize them for general wellness and addressing specific health reasons, particularly mental health^13–16^. Thus, at least some contemplative practices (mindfulness, yoga) may be well-suited to mental health prevention, early intervention, as well as acute and chronic illness management.

Contemplative practices are increasingly popular in countries across the world. Recent data from nationally representative surveys suggests lifetime prevalence rates for meditation from 11.4% in India^17^ and 15.1% in Germany^14^ to 18.3% in the US^8^. For yoga, lifetime estimates range from 15.1% in Germany^18^ to 16.8% in the US^8^ and 16.9% in India^17^. Similar rates have been found for the 12-month prevalence of these practices in Iceland^19^ and Norway^20^. Tai chi/ qigong and visualisation/ guided imagery rates have been generally lower at around 3% in the US^21^ and Norway^20^. In general, the prevalence, accessibility and acceptance of contemplative practices have grown consistently over recent years.

A previous publication on data from this representative survey of Australian and New Zealand populations indicated a very high lifetime prevalence of meditation practice in these two countries, 41.5% and 35.7% respectively^22^. Meditators in both countries were found to be younger, female, more educated, indigenous, LGBTQIA+, have worse mental health and higher unmet need for mental health care, and more likely to utilise complementary medicine or mental health care. It remains unclear whether meditation users have worse mental health as a result of engaging with these offerings or if other sociodemographic (e.g., socioeconomic status) and behavioural (e.g., physical activity, alcohol and substance use, mental healthcare use) patterns between users and non-users account for differences in mental health.

The current paper extends these previous findings on the same data set by addressing two other pre-registered questions (osf.io/etkh4) and conducting explorative posthoc analyses to further clarify the previously established relationships. Thereby, we expand the focus from meditation to contemplative practices in general and examine their prevalence and mental health associations across Australia and New Zealand. As contemplative practices encompass a wide range of practices, we first establish how participants interpret this label and which practices are most prevalent in our sample (pre-registered question 4). Then, we explore potential differences between practitioners of the most prevalent contemplative practices with respect to sociodemographic and individual factors, mental healthcare use, and psychological distress (pre-registered question 3), and explore reasons for engaging in contemplative practices. Finally, we conduct posthoc regression analysis to investigate how patterns between contemplative practice use and psychological distress change when we account for previously observed differences between practitioners and non-practitioners. By answering these questions, we hope to contribute to an ongoing discussion on the utilization and suitability of different contemplative practices for addressing growing mental health needs.

## Methods

### Participants

The sample comprised 2,640 individuals from Australia (*n* = 2,069) and New Zealand (*n* = 571). The sample was nationally representative per quota for age, gender, ethnicity, state/territory, and income bracket (see^22^. Overall, there were 52.5% women in the sample, the mean age was 46.8 years (*SD* = 17.9), 40.2% of participants had a university degree, 13.9% identified as LGBTQIA+, and 18.1% were of indigenous descent. Further details on sample characteristics are presented in the results section and are available at the Open Science Framework (https://osf.io/j3hta).

### Study design

The data for this study were obtained from a cross-sectional online survey conducted from 27 September 2023 to 8 January 2024. Survey responses were self-reported and gathered through Qualtrics’ panel aggregation system which sources participants from multiple online panels to enhance demographic diversity. All participants provided written informed consent and were reimbursed for their time. The study was approved by University of Melbourne Human Research Ethics Committee (#23004) and was performed in accordance with relevant guidelines and regulations. More methodological details can be found in a previous publication^22^. The study protocol was preregistered (https://osf.io/etkh4). The current analysis focuses on the third and fourth preregistered research questions that were not covered in the initial report. Two modifications/deviations from the preregistration were made. (a) To improve generalisability in question 3, we compared meditators to the three contemplative practices that were comparably prevalent in the sample (relaxation, breathing techniques, yoga) rather than merely yoga. (b) To be able to analyse the complete data set including both Australian and New Zealand data, some region-specific covariates had to be dropped (ethnicity, remoteness, socioeconomic status). The ethnicity questions for Australia and New Zealand had distinct categories that could not be combined. Remoteness and subsequently socioeconomic status related to postcode could not be calculated for New Zealand. In any case, we have included income, education and employment status in the analysis. These variables capture several aspects of socioeconomic status across the two countries without relying on postcode.

### Measures

The survey contained 76 items assessing contemplative practice use, sociodemographics, faith and spirituality, health behaviours, and mental health.

### Contemplative practice

To determine practice prevalence and engagement, participants received a block of general questions pertaining to the following: Q1) Any lifetime contemplative practice use (yes/no); Q2) Lifetime practice: Specific types of lifetime contemplative practice (multiple choice from pre-specified list: meditation, yoga, relaxation, guided imagery, tai chi, qigong, breathing exercises, visualization, other contemplative practices); Q3) Option to describe any other contemplative practice used, apart from those already listed (open response); Q4) Past-year practice: Use of specific contemplative practices in past 12 months (same multiple choice list); Q5) Main practice in the past year: Indication of which practice, among those used in past 12 months, was the main practice. Notably, we did not provide definitions for any of the contemplative practices listed in the survey. Participants could select ‘meditation’ or any other practice based on their own judgement. This enabled us to capture all forms of contemplative practices as defined by practitioners rather than researchers.

Participants’ responses to Q4 formed the basis of the subsequent block of detailed questions regarding their contemplative practice. If participants selected multiple practices in Q4, they were asked subsequent questions about the practice that came first in this list: meditation, yoga, relaxation, guided imagery, tai chi, qigong, breathing exercises, visualization, other contemplative practices. This list is ordered from most to least prevalent based on prior estimates of use rates. In addition, meditation practice was prioritised in this survey to permit addressing pre-registered questions about meditation (see^22^. This approach allowed us to examine all participants who practiced meditation, even if it wasn’t their primary practice. A major limitation of this approach, however, is the lack of full responses for any other contemplative practice, and inconsistencies between a participant’s actual main practice and the practice for which they answered questions (assigned practice). These inconsistencies applied to 24.8% (*n* = 457) of cases (see Supplementary Figure A1 for a full breakdown). Questions in this block included participants’ specific style of practice, their practice motivation and context, and specific techniques they used. Due to the limitations above, we restricted our analyses of questions from this block (i.e., styles, motivation, context, techniques) to providing high level summaries.

### Sociodemographic and individual variables

Sociodemographic variables included in this paper were gender, age, education, income, employment status, disability status, chronic disease status, LGBTQIA+ identity, and indigenous identity. Faith and spirituality were assessed by questions evaluating participants’ connectedness to humanity, nature, and a higher power, their degree of spirituality and religiosity, and their current religious affiliation. Health behaviours included physical activity (days per week), alcohol, tobacco, cannabis, and psychedelic drug use. Mental healthcare utilisation captured appointments with mental health professionals (e.g. psychologist, counsellor) in the past 12 months, with response options capturing having used or not used these services as well as unmet need. Although utilisation of other healthcare services (medical or complementary) was collected in the survey, it was not the primary focus of this paper and hence not included. Detailed analyses and results on all healthcare services have been previously reported^23,22^. To increase simplicity and statistical power, we simplified or dichotomized several variables for use in this paper (see Supplementary Table A2). For example, questions on chronic disease or carer responsibilities had multiple response options, which we collapsed into a single category (any disease or any caring role). Similarly, education was collapsed to having a university degree or not, and occupation into working or not working. Responses on religion, spirituality, and substance use were simplified to no/not at all/never versus any affirmative response.

### Psychological distress

The primary outcome for this paper is psychological distress as measured by the 6-item Kessler Psychological Distress Scale (K6^24^. The K6 is a widely used and well validated measure of psychological distress and a screening tool for serious mental illness in the general population^25^. Items were scored on a 5-point Likert scale (0 = *none of the time* to 4 = *all of the time*) and a total score ≥ 13 is considered indicative of serious mental distress. The reliability of the K6 in this sample was very high, α = 0.92.

### Data analysis

We used descriptive statistics and visualisation techniques to explore the prevalence of contemplative practices over different time frames, the motivations for engaging in these practices, and the relative mental healthcare use across practices. Correlation analysis was used to investigate which practices were commonly practiced together. For open-ended responses, we followed the process of qualitative content analysis^26^ to categorize and count responses in an iterative and data-driven process. We first deducted codes closely matching participants’ responses, and then reiterated and reorganised codes to capture thematically similar categories. Finally, we grouped the emergent categories into meaningful clusters of related concepts and counted the number of responses in each category and cluster. Differences between contemplative practices regarding sociodemographic and individual variables were summarized using descriptive statistics. Between-group comparisons utilized analysis of variance (ANOVA) for continuous data, Kruskall-Wallis tests for ordinal data, and Chi-Square for categorical data. False Discovery Rate (FDR) adjustment was used to correct *p*-values for multiple testing.

We explored differences with respect to psychological distress by conducting two complementary analyses. First, we compared the distribution and mean score of psychological distress across non-contemplatives and the four main groups of contemplative practitioners utilizing ANOVAs and post-hoc tests. This analysis used participants’ main practice as a grouping variable. Second, we conducted regression analysis predicting distress by contemplative practice use in the past 12 months. This analysis included all participants who had engaged in a specific practice in the past 12 months, irrespective of whether it was their main practice. We report similar regression analyses with a different approach to grouping contemplative practice use on quality of life elsewhere (Bailey et al.,^23^).

We estimated four regression models for each of the four most prevalent contemplative practices. First, we predicted psychological distress by having practiced one of these contemplative practices in the past 12 months (Model 1). In a second step, we included all relevant sociodemographic and individual variables (except healthcare use) as predictors to statistically control their influence in multiple regression analyses (Model 2). To reduce the number of covariates and thus Model 2 complexity, first we performed regression analyses with psychological distress as dependent variable and all covariates as predictors, and then retained all factors that were significant and excluded non-significant ones. In the final two models, we added mental healthcare use (Model 3) and its interaction with contemplative practice (Model 4). If there was a significant interaction, we estimated marginal means and performed *t*-tests comparing practitioners and non-practitioners across the three healthcare use categories.

All statistical analyses were performed using R 4.4.3^27^. All data, scripts, and materials that support the results can be found at https://osf.io/4jzwf.

## Results

### General prevalence

Table 1 summarizes the overall prevalence of different practices over lifetime (Q2), the past 12 months (Q4) and the self-identified main practice in the past 12 months (Q5).

**Table 1:** Overall prevalence of different practice types during lifetime and past year, as well as main practice in past year for total sample (*N* = 2640).

Practice	Lifetime	Past year	Main past year
	% (*n*)	% (*n*)	% (*n*)
Meditation	40.3 (1063)	31.1 (820)	19.5 (512)
Relaxation techniques	35.0 (924)	25.4 (670)	11.2 (295)
Breathing techniques	34.0 (897)	24.4 (643)	13.9 (365)
Yoga	30.9 (815)	21.4 (564)	14.3 (375)
Visualisation	14.7 (389)	7.3 (192)	2.1 (56)
Guided Imagery	10.6 (281)	4.9 (129)	1.7 (44)
Tai Chi	10.9 (288)	4.7 (125)	2.4 (63)
Qi Gong	5.8 (146)	2.0 (53)	0.9 (26)
Other contemplative practice	5.5 (154)	4.4 (117)	3.8 (99)
None of these	30.2 (798)	30.1 (794)	30.2 (794)
Total	218.0 (5755)	155.6 (4107)	100 (2629)

The most common contemplative practice across all three questions was meditation (lifetime 40%, 12-month 31%, main 20%) followed by relaxation, breathing techniques and yoga with a lifetime prevalence between 31 and 35% and a 12-month prevalence between 21 and 25%. Visualisation, guided imagery, Tai Chi, Qi Gong and other contemplative practices were less common with a lifetime prevalence between 6 and 15% and a 12-month prevalence between 2 and 7%. Supplementary Figure B1 gives a visual impression of the percentages with which the four main practices were chosen during lifetime and past year, with additional consideration of main practice.

Of all participants who engaged in any contemplative practice, 74.9% reported having practiced more than one contemplative practice during their lifetime and 45.1% reported more than one in the past 12 months. Meditation, breathing, and relaxation tended to be co-utilised, while visualisation was linked to breathing, imagery, and relaxation. Further results on which practices tended to be practiced together can be found in the Supplementary Section B2.

### Interpretation of the term “contemplative practice”

**Fig. 1:**
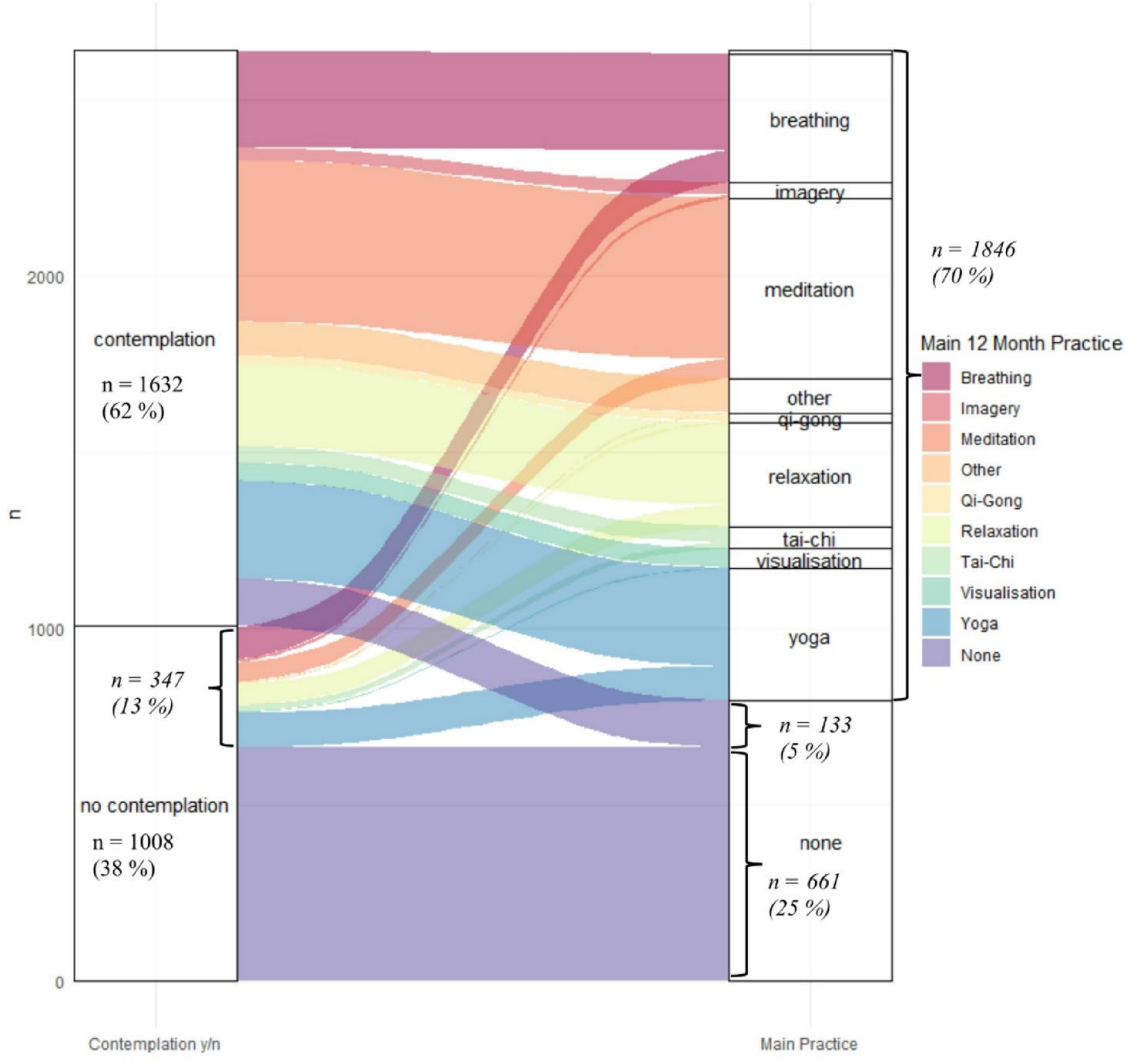
Response flow between lifetime contemplative practice use into self-reported main practice in the past year. Displayed percentages refer to total sample size.

Figure 1 provides a detailed breakdown of response flow between lifetime contemplative practice use (Q1) and past year main practice (Q5).

The majority of participants (61.8%) indicated they had used a contemplative practice in their lifetime. However, 34.4% (*n* = 347/1008) of participants who denied a lifetime contemplative practice in the simple yes-no question, reported a past-year practice when presented with a list, most commonly yoga or breathing. Only 8.2% (*n* = 133/1632) of those who reported a lifetime contemplative practice did not endorse a past-year practice. Taken together, these findings suggest participant interpretation of the generic term “contemplative practice” that was potentially at odds with the research team, even though the question provided a definition and a few examples of contemplative practices. Presenting participants with a list of practices seemed to circumvent this problem.

Of the total 1,846 individuals who endorsed use of a contemplative practice in the past year, 60.5% denied use of a practice other than those listed. Among the other 39.5%, we categorized the 729 open response entries in an iterative process. Of these, 16.3% (301/1842) were classified as reiterations of previously listed practices; another 13.5% (250/1842) were classified as alternative, unlisted practices; 2.4% (44/1842) were classified as motivations rather than contemplative practices (e.g., reduce stress, healing); and 7.3% (134/1842) could not be classified (e.g., ponder, pure heart, meaningful). Among the alternative, unlisted categories of contemplative practice, walking (3.5%, *n* = 65), prayer (3.1%, *n* = 57), gardening (2.3%, *n* = 42), and different forms of sports or exercise (1.8%, *n* = 33) were mentioned most (see Supplemental Table C1 for a full list of categories). Taken together, open responses suggested lifetime use of established practices by 76.8% of those engaging in contemplative practices, with the remaining 23.2% engaging in possibly less well defined or described practices.

Pertaining to pre-registered question 4, we analysed participants’ style of contemplative practice (see Supplementary Section C for detailed results). Among meditators, only 7% of participants named a tradition (most commonly yoga), while 43% described a technique (most commonly breath), 13% described a mode rather than style of practice (most commonly guided), and 16% named a motivation for practice (most commonly to relax; see Supplementary Table C2). We further explored how meditators were practicing and found the most used meditation techniques were body-centered meditation and mindful observation, and the most common practice contexts were practicing alone, self-guided, or listening to audio or video resources. Overall, participants practiced in a wide range of contexts and used various meditation techniques (see Supplementary Tables C3 and C4).

Among all other contemplative practices, the most common category in a given practice was the unspecific ‘other’ category (see Supplementary Table C5). Descriptions of breathing practices were common among breathing, relaxation and yoga practitioners, most commonly falling under the category of ‘other’ or ‘deep breathing.’ As all categories were derived from the data, this indicates a rather broad and varied interpretation by participants (and not necessarily a conceptual framework) of both the different contemplative practices and their specific styles. Moreover, the rather nonspecific question asking for ‘style of practice’ might have contributed to this result.

### Sociodemographic and individual differences

As previous research indicated substantial sociodemographic and individual differences between meditators and non-meditators^22^, we next compared practitioners of the four main contemplative practices and non-contemplatives on a range of these factors (as outlined in the preregistration). Table 2 provides descriptives across the whole sample and in each of the five groups. Participants were grouped by their main practice. Supplementary Section E details the full demographic descriptives for each subgroup prior to simplification and includes region-specific variables (income, ethnicity, remoteness).

**Table 2:**
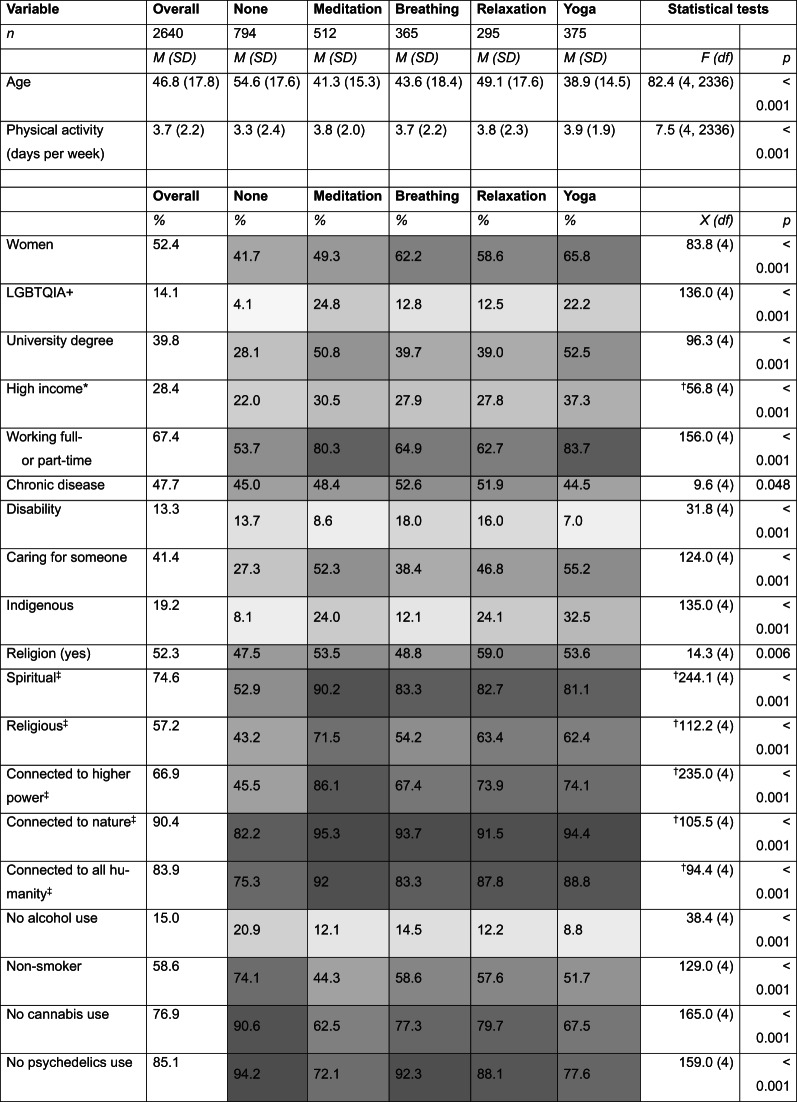
Sociodemographic and behavioural descriptives by sample and subsampling of contemplative practice groups.

We split some ordinal variables into two categories to simplify reporting and shaded the subgroups from light (low percentage) to dark grey (high percentage) to ease interpretation. * = High income in AU: >$156,000 and NZ: >$70,000; ^†^ = Kruskall-Wallis Chi-Square; ^‡^ = all values higher than “not at all”.

Table 2 reveals significant differences in all sociodemographic and individual factors between the five groups. The most notable differences were between non-contemplatives and contemplatives. Non-contemplatives were generally older, less physically active, less religious, and felt less connected to a higher power, nature or humanity. They were less likely to be women, carers, people of indigenous descent, identify as LGBTQIA+, have a university degree or high income, or be working. They were less likely to use alcohol, engage in smoking, or use cannabis or psychedelic drugs.

Among contemplatives, yoga practitioners and meditators were more likely (than other contemplatives) to be younger, have a university degree, be carers, be working, identify as LGBTQIA+, use cannabis or psychedelics, and were less likely to have a disability. Meditators were more spiritual, religious, and connected to a higher power, and had a more equal gender ratio than the other three contemplative practices. Yoga practitioners were more likely to be of indigenous descent, have a higher income, drink alcohol, and less likely to have a chronic disease. Relaxation practitioners were more likely to follow a religion.

### Why do people practice contemplation?

**Fig. 2:**
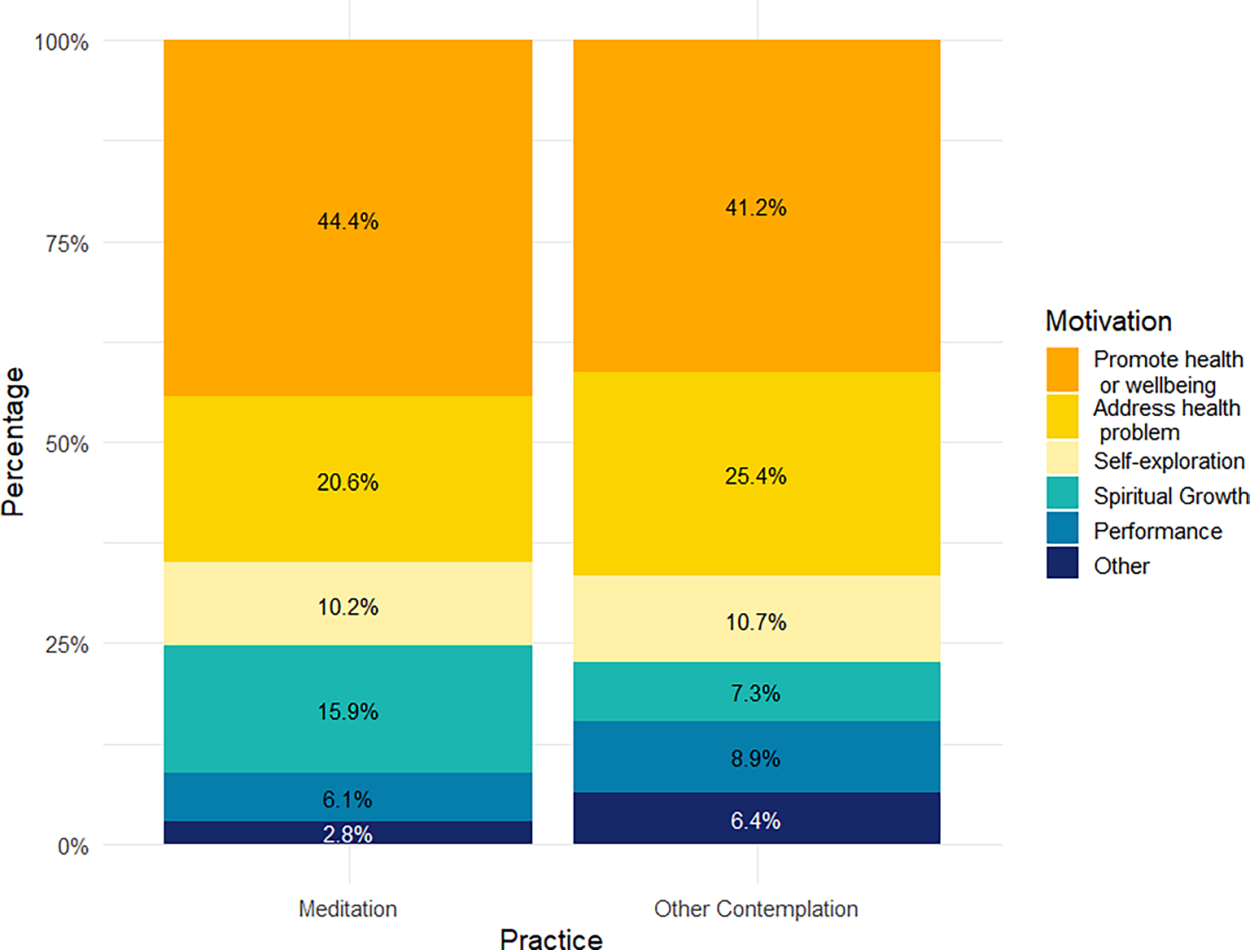
Motivations for starting meditation or other contemplative practice.

Participants were asked to select from among six reasons for starting a contemplative practice. The most popular reasons for starting a given contemplative practice were promoting health or wellbeing (42.6%) and addressing a health problem (23.3%), followed by spiritual growth (11.1%), self-exploration (10.5%), performance (7.6%), and other (4.8%). Figure 2 shows a comparison of relative endorsement of these reasons for meditators and other contemplation practitioners.

Across both groups, promoting wellbeing and health was reliably the largest motivation. A global Chi-Square test indicated significant differences between the groups, *Χ*^2^(5) = 52.7, *p* < 0.001. Subsequently, we performed individual Chi-Square tests comparing each motivation across groups and corrected the resulting *p*-values using FDR-adjustment. There were significant differences between groups for two motivations – meditators were motivated more to practice for spiritual growth (*Χ*^2^(1) = 32.8, *p* < 0.001) and less for addressing a health problem (*Χ*^2^(1) = 5.7, *p* = 0.043). The other motivations were not significantly different between groups – promoting health or wellbeing (*Χ*^2^(1) = 1.7, *p* = 0.241), self-exploration (*Χ*^2^(1) = 0.1, *p* = 0.798), and performance (*Χ*^2^(1) = 4.6, *p* = 0.054).

### Mental healthcare use

**Fig. 3:**
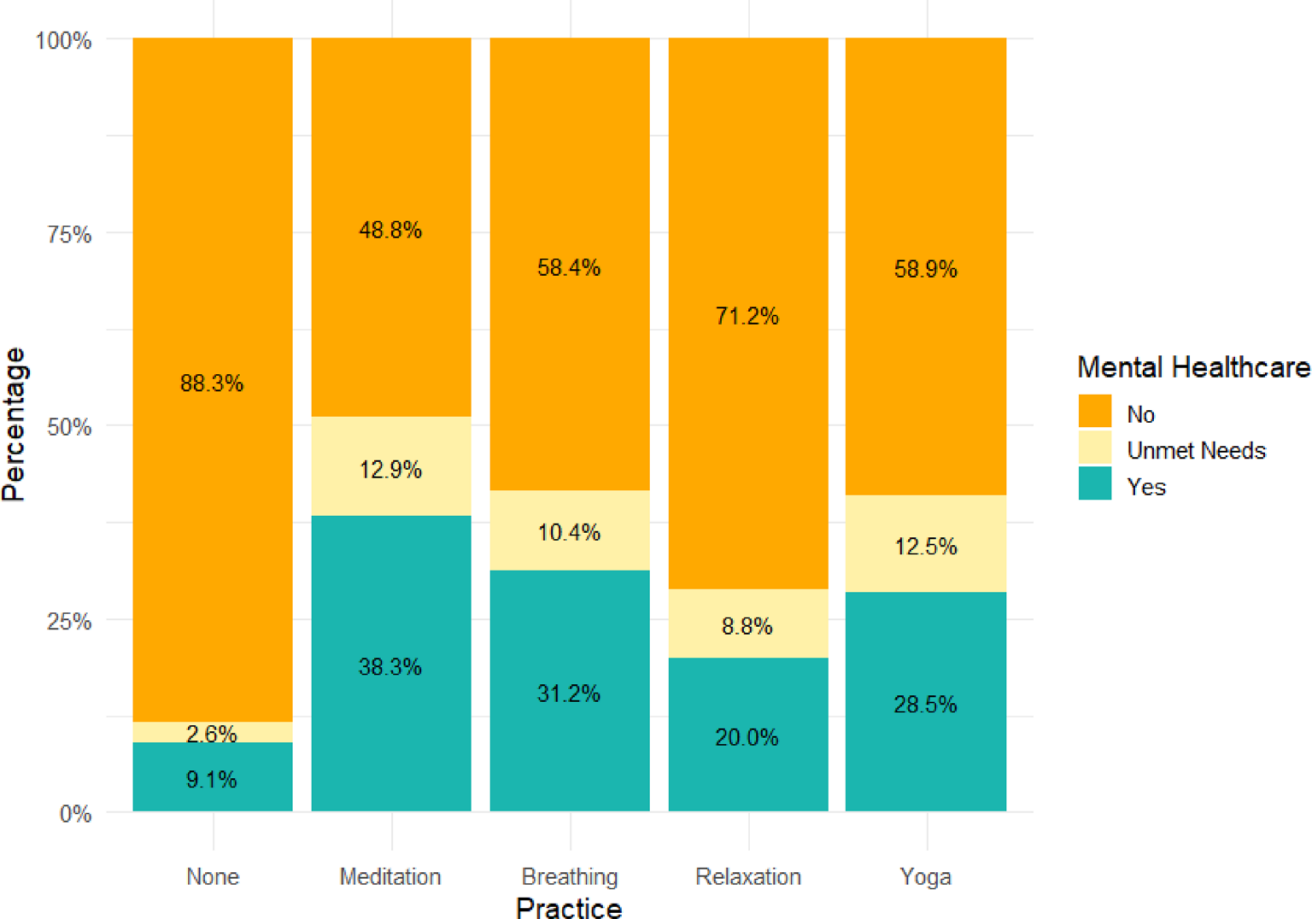
Relative mental healthcare use and unmet needs by main contemplative practice.

Next, we compared mental healthcare use between the four most common contemplative practices and those who did not engage in any practice. Figure 3 shows participants’ mental healthcare use and level of unmet needs.

Participants across all four contemplative practices report considerably higher use of mental healthcare and higher levels of unmet needs compared to non-contemplatives, with the highest proportions in meditators (12.9%). A global Chi-Square test indicated significant differences between the groups, *Χ*^2^(8) = 271.6, *p* < 0.001. Individual Chi-Square tests with FDR adjustment revealed significant differences in healthcare use for non-contemplatives compared to all contemplative groups (*Χ*^2^(2) = 225.2, *p* < 0.001), with lower rates of mental healthcare use and unmet need in the former. Within contemplative groups, meditators had higher mental healthcare use and unmet need than other contemplative practitioners, (*Χ*^2^(2) = 26.1, *p* < 0.001), while relaxation users had lower mental healthcare use and unmet needs, (*Χ*^2^(2) = 27.3, *p* < 0.001). Breathing (*Χ*^2^(2) = 0.5, *p* = 0.778) and yoga practitioners (*Χ*^2^(2) = 1.4, *p* = 0.611) were no different to other contemplative practitioners.

### Relation of contemplative practices to psychological distress

#### Differences by main practice

**Fig. 4:**
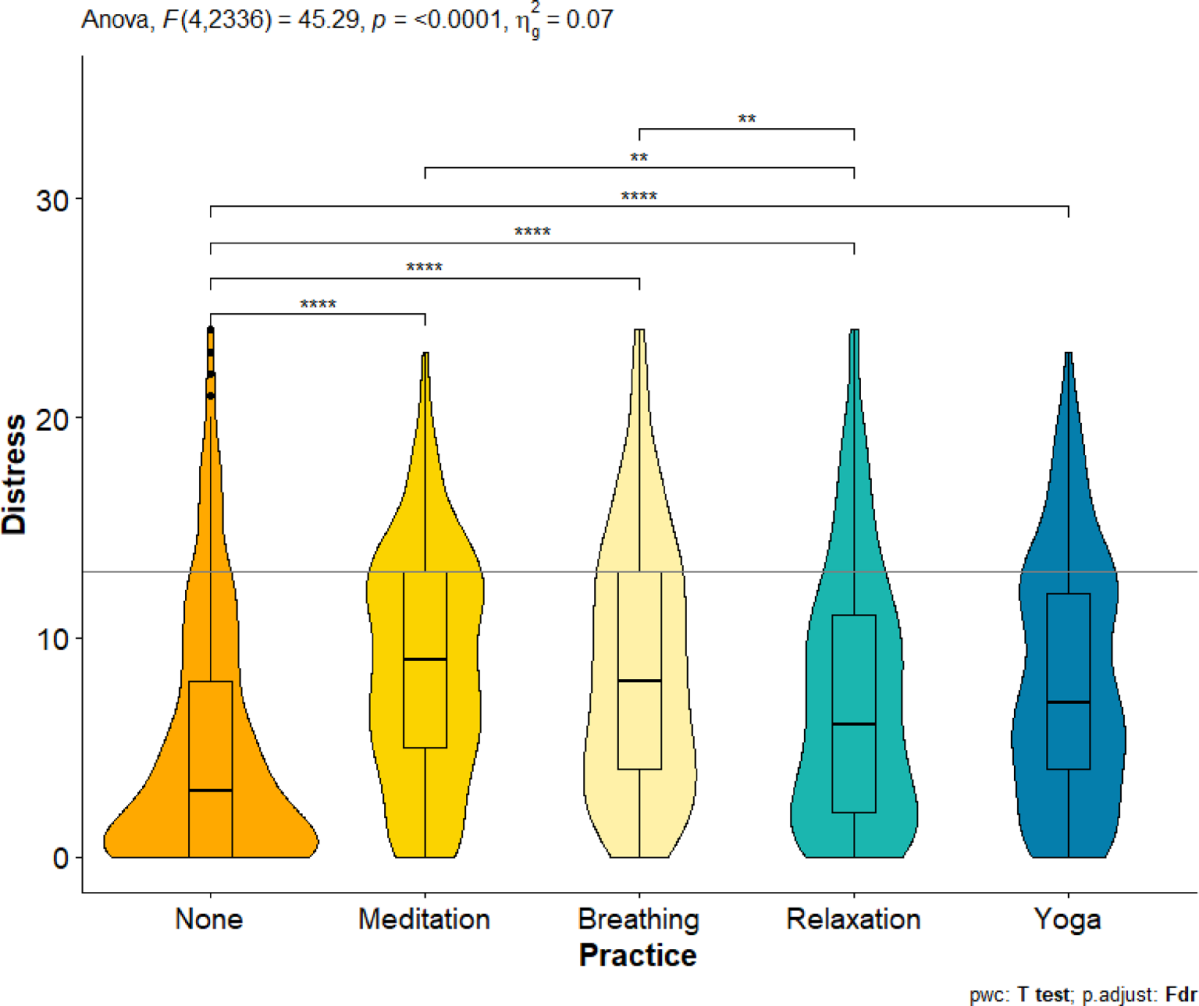
Psychological distress across participants by main contemplative practice. A thin horizontal line was drawn at the cut point = 13, indicating likely serious mental illness (SMI). Box indicates 25th to 75th percentiles, middle bar corresponds to median, and whiskers represent the largest and lowest values within 1.5 times the interquartile range. Asterisks indicate significant pairwise comparisons: ** *p* < 0.01, **** *p* < 0.0001.

The average psychological distress score in this sample was *M* = 7.26 (*SD* = 5.82), indicating low to moderate distress. There was a statistically significant difference between the five groups on K6 scores, *F*(4,2336) = 45.3, *p* < 0.001 (see Fig. 4). All four contemplative groups reported significantly higher levels of distress than the no-practice group (*p* < 0.05 with FDR correction). Among contemplative practitioners, those who used relaxation reported the lowest levels of distress, a difference that was only significantly different to meditation and breathing (see Supplemental Table D1 for all comparisons).

#### Differences by practice in the last 12 months

As there were significant sociodemographic and mental healthcare utilisation differences between contemplative practice groups, we next explored whether those features could account for variation in psychological distress within the four main practice groups. The following tables display the regression estimates for psychological distress predicted by meditation practice, relaxation practice, breathing practice, and yoga practice in the past 12 months (see Table 3). Model 1 includes only the respective contemplative practice as a predictor; Model 2 adds all relevant covariates (see Supplementary Table D2 for covariate reduction model); Model 3 further adds mental healthcare use; and Model 4 includes an interaction term for healthcare and contemplative practice. Details for full models including covariates are provided in Supplementary Section D (Tables D3 to D6).

**Table 3:** Standardised regression parameter estimates of psychological distress on contemplative practices, sociodemographics, and mental healthcare use.

	Model 1	Model 2	Model 3	Model 4
	β	β	β	β
Meditation	0.164^***^	0.041^**^	0.008	0.031
Mental HC (unmet)			0.163^***^	0.209^***^
Mental HC (yes)			0.192^***^	0.194^***^
Meditation * Mental HC (unmet)				-0.073^***^
Meditation * Mental HC (yes)				-0.011
*R* ^*2*^_*adj*_	0.026	0.325	0.365	0.367
Relaxation 12-month	0.075^***^	0.019	0.004	0.007
Mental HC (unmet)			0.163^***^	0.190^***^
Mental HC (yes)			0.193^***^	0.181^***^
Relaxation * Mental HC (unmet)				-0.049^*^
Relaxation * Mental HC (yes)				0.021
*R* ^*2*^_*adj*_	0.005	0.324	0.365	0.367
Breathing 12-month	0.110^***^	0.073^***^	0.051^**^	0.042^*^
Mental HC (unmet)			0.161^***^	0.164^***^
Mental HC (yes)			0.188^***^	0.176^***^
Breathing * Mental HC (unmet)				-0.006
Breathing * Mental HC (yes)				0.024
*R* ^*2*^_*adj*_	0.012	0.328	0.368	0.368
Yoga 12-month	0.092^***^	-0.026	-0.033	-0.04
Mental HC (unmet)			0.165^***^	0.171^***^
Mental HC (yes)			0.194^***^	0.185^***^
Yoga * Mental HC (unmet)				-0.011
Yoga * Mental HC (yes)				0.021
*R* ^*2*^_*adj*_	0.008	0.324	0.366	0.366

Model 1 includes only contemplative practice. Model 2 includes sociodemographic covariates. Model 3 includes mental healthcare. Model 4 includes interactions between contemplative practice and mental healthcare. Reference condition for Mental HC is *‘no’*. Reference condition for interaction is contemplative practice x Mental HC (no). HC = healthcare; *p < 0.05; **p < 0.01; ***p < 0.001.

All four contemplative practices were significantly associated with psychological distress (Model 1). When sociodemographic and individual covariates were added, there was no longer a significant association for relaxation and yoga with psychological distress, however, meditation and breathing still showed significant associations. Including mental healthcare use eliminated the association of meditation but not breathing with psychological distress. There were significant interactions of mental healthcare use with meditation and relaxation in relation to psychological distress.

**Fig. 5:**
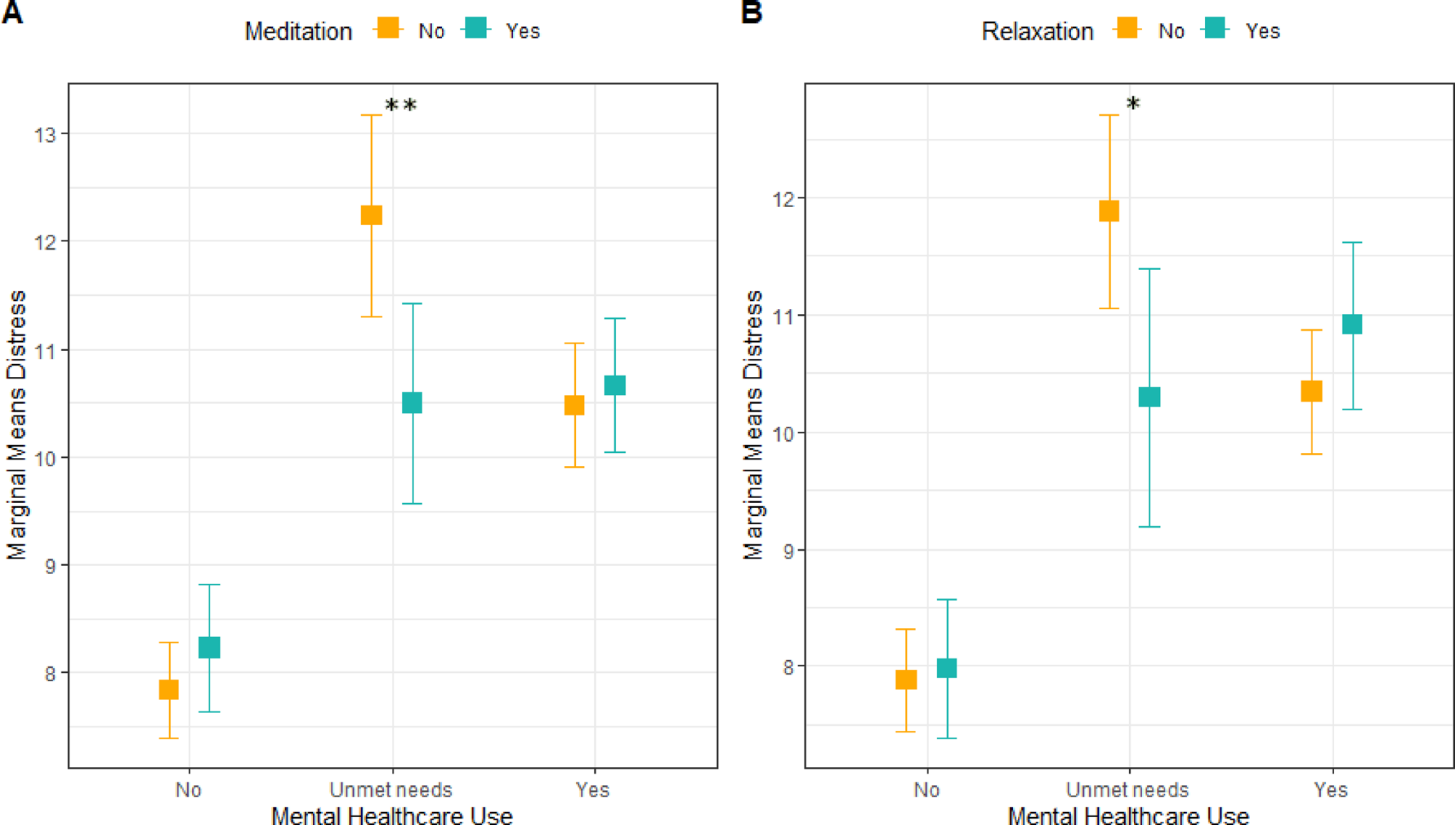
Estimated marginal means for distress scores across (**a**) meditation or (**b**) relaxation practice in the past 12 months and use of mental healthcare. *p < 0.05; **p < 0.01.

Post hoc pairwise comparisons for distress revealed a significant difference between meditators (*M* = 10.5, [95%-CI 9.6; 11.4]) and non-meditators (*M* = 12.2 [11.3; 13.2]) for unmet healthcare needs, *t*(2577) = 1.74, *p* = 0.006, *d* = 0.38, but not for having used or not used healthcare. Post hoc tests revealed significantly lower distress among relaxation practitioners (*EMM* = 10.3, [95%-CI 9.2; 11.4]) compared to non-practitioners (*EMM* = 11.9 [11.1; 12.7]) with unmet healthcare needs, *t*(2577) = 1.59, *p* = 0.017, *d* = 0.34, but not for those having used or not used healthcare. Figure 5 displays estimated marginal means for both interactions.

## Discussion

In this nationally representative survey of Australian and New Zealand populations, approximately 70% of the sample reported use of a contemplative practice in the past year, most commonly meditation, relaxation, breathing techniques, and yoga. Contemplative practice use was associated with sociodemographic indicators of disadvantage regarding health equity (e.g., female gender, LGBTQIA+ status, indigenous descent), as well as those related to higher healthcare access (more education, higher income, more employment). Promoting health or wellbeing and addressing a health problem were the main reported reasons for people using contemplative practices and these individuals had higher use and unmet need of mental healthcare. Notably, contemplative practitioners had, on average, more psychological distress. For yoga and relaxation, sociodemographic variables fully explained this association. For meditators, mental healthcare use explained the association. For those using breathing techniques, the effect remained significant even after controlling for covariates. However, among those with unmet mental healthcare needs, those who used meditation or relaxation had less psychological distress. Taken together, these results suggest that people might be using contemplative practices to manage their mental health. However, as our data is cross-sectional, we cannot conclude whether these practices have a causal link to mental health; people might be using contemplative practices to manage psychological distress, but contemplative practice could also be contributing to psychological distress.

### Contemplative practices are popular

Rates of contemplative practice use were consistently higher than previously reported in Australia^28,29^ and other countries^14,8,20,21,17,19^. This result is in line with increasing practice rates across countries and could, in part, reflect the detailed methodology of the present study that explicitly investigated 8 plus contemplative practices. Nonetheless, Australian and New Zealand adults exhibited an exceptionally high level of engagement with contemplative practices compared to other countries. Compared to the to-date most reliable estimates (from the US National Health Interview Survey^8^, 31.1% of Australians and New Zealanders practiced meditation in the past year (US: 18.3%), 25.4% practiced relaxation (US: 6.7%), 24.4% practiced breathing techniques (US: not available), and 21.4% practiced yoga (US: 16.8%). Upwards of three quarters of those endorsing contemplative practice used a known contemplative practice, providing further confidence in our estimates.

The most prevalent contemplative practice was meditation, followed by relaxation, breathing techniques and yoga. Other contemplative practices were less common, used in the last year by less than 8% of the sample. This pattern is similar to those observed in other countries with meditation, yoga and relaxation being the most common practices in the US, Germany and Norway^14,18,8,20^. Breathing practices seem to be less researched and less common^30^, except among yoga practitioners^17,31^. Often, participants engaged in multiple practices at the same time, suggesting that contemplative practices are likely part of a broader strategy to manage health and promote wellbeing.

Practitioners of various contemplative practices differed both from non-practitioners and from each other with respect to sociodemographic factors, spirituality and health behaviours. These findings align with previous research showing that individuals who practice contemplative practices tend to be female, younger, higher earning and more educated compared to non-practitioners^32,19^, while also showing poorer health^8,33^. This pattern is similar to complementary and alternative medicine (CAM) users in general^34,35,29^. At the same time, contemplative practice users were more likely to be carers and identify as LGBTQIA + or Indigenous – these factors being commonly associated with poorer mental health^36,37^. While use of contemplative or CAM practices is generally associated with a healthier lifestyle^38–40^, a large survey of Australian women showed higher use of marijuana and illicit drugs among those who practiced meditation or yoga^41^, and US adults using meditation, yoga, relaxation or tai chi/qigong were more likely to drink alcohol than non-users^8,21^. The higher spirituality observed among meditators is consistent with the spiritual foundations of many meditation traditions^42^. Thus, contemplative practices tend to attract people from both advantaged and disadvantaged social groups.

This seemingly divergent pattern might, on the one hand, reflect different groups of people utilising these practices for different reasons. Individuals experiencing higher levels of need and distress might engage with them to address unmet needs, whereas younger and more affluent users might incorporate them as part of a broader healthy lifestyle. On the other hand, these sociodemographic categories are not mutually exclusive and may intersect as, e.g., a queer woman with a university education fits both profiles. Similarly, high socioeconomic status minority individuals might benefit from the emotion-regulation and identity-affirming effects of contemplative practices when facing minority stress.

### People use contemplative practices for mental health

Improving health and wellbeing were the most common reasons for starting a contemplative practice, corresponding with earlier findings^14,15,16,43^. Interestingly, meditators had a stronger motivation for spiritual growth and lower motivation for addressing a health problem than other contemplative practitioners, consistent with prior findings that meditation is more likely to be sustained for reasons beyond improving mental health^44^. Nevertheless, alongside numerous other reasons, mental health seems to be particularly important for beginning a meditation or yoga practice^13,45–47^. This might in part be the result of public perception, marketing and referral of these practices. Contemplative practices are often advocated as viable means to improve public health^48,49^. Surveys among clinicians indicated strong endorsement of these practices – 65% and 62% of Australian GPs recommended meditation and yoga, respectively^50^, 90% of Australian psychologists recommended mind-body approaches^51^, and 71% of US physicians recommended meditation to their patients^52^. Numerous studies support the positive effects of these practices on mental health and stress reduction.

In contrast to this common perspective, prior data on meditation, yoga, guided imagery, and relaxation among US adults showed that these practices were linked to greater use of mental health services and greater psychological distress^8^. In prior analysis of the current data, we also found that meditation was related to decreased quality of life^22^. Together, these findings might suggest that unwell people are engaging in contemplative practices to manage their health, or that these practices harm people’s health^53,54^, or some combination. The present analysis explored whether associations between contemplative practice and psychological distress might be accounted for by sociodemographic factors and/or mental healthcare use. Practitioners of the four most commonly used contemplative practices (meditation, relaxation, breathing, yoga) reported higher rates of mental healthcare use and unmet need than non-practitioners. Our results are consistent with prior work, which found that both health problems and healthcare access and cost barriers predicted contemplative practice use^55^. Furthermore, all contemplative groups exhibited higher levels of psychological distress than non-contemplatives.

When we controlled for sociodemographic and individual differences, relaxation and yoga were no longer associated with increased distress, suggesting that factors such as low income, disability or chronic disease status may better explain associations of these practices with psychological distress. These factors have been previously identified as risk factors for distress^56^ and unmet healthcare need^57^. In other words, individuals with multiple presenting challenges are likely to have greater psychological distress and also to use yoga and relaxation, possibly in an effort to mitigate the impact of their situation. US survey respondents reported using alternative medicine (particularly self-care therapies such as contemplative practices) instead of conventional care either due to cost/non-cost barriers or because they did not find conventional care helpful^58^.

Controlling for mental healthcare use eliminated the association with meditation, indicating meditation may be commonly used alongside or complementary to mental health treatment. Representative surveys indicate higher rates of mental healthcare use among meditators^8^, and many clinicians recommend mindfulness and meditation to their patients^52,50^. It is important to acknowledge that globally, mental health services have limited accessibility and even in good circumstances are often not adequate^3^. Meditation and other contemplative practices might thus provide additional mental health benefits, specifically where there is unmet need. This corresponds with our finding that meditators and relaxation users with unmet need reported lower distress than non-practitioners with unmet need with effect sizes of *d* = 0.35–0.38, suggesting these practices may serve as a potentially helpful means to manage mental health when traditional care is inaccessible. More research is needed to explore this possibility.

Breathing practices remained associated with psychological distress even when accounting for sociodemographic factors and mental healthcare use, indicating a potentially ineffective use of these practices. Breathing practices or breathwork is an emerging field of research with a recent meta-analysis establishing small-to-medium reductions in stress, anxiety and depression^59^. However, a recent review outlined several factors that contribute to the effectiveness of these practices, including slow-paced rather than fast-paced breathing, multiple sessions, longer duration of both breathing sessions and interventions, and human-guided training^60^. Our study provides little detail on how participants practiced breathing, the most common response being deep breathing. Thus, participants might have been using less effective versions of breathing practice than necessary for a notable reduction of distress. There are still considerable gaps in understanding not only the efficacy of contemplative practices, but also their effectiveness, dissemination and implementation on a larger scale^61,62^.

### Limitations and Implications

The present study provides an in-depth examination of contemplative practice use and mental health correlates in a nationally representative sample of Australians and New Zealanders. Although the sample was representative, the online data collection might have introduced selection bias and under-representation of participants who had limited internet access. The survey did, however, use a generic title (‘National Wellness Survey’) and did not include any specific information about its contents to avoid self-selection of contemplative practitioners.

The cross-sectional nature of our data precludes us from making causal inferences. We cannot discern whether participants experience psychological distress because of or despite using contemplative practices, nor does our data support statements of efficacy regarding reductions in distress. More methodologically rigorous studies (e.g., prospective longitudinal studies, randomised controlled trials) are needed to examine the safety and efficacy of various contemplative practices. We could, however, demonstrate that meditation, relaxation and yoga were no longer associated with increased distress after controlling for all differences found between users and non-users of these practices. We also found links to reduced psychological distress among meditation and relaxation users with unmet mental healthcare needs, suggesting that contemplative practices may play a complementary and/or alternative role in managing mental health.

The wide variety of contemplative practices, schools and traditions makes it challenging to draw conclusions for a given practice. Most participants reported genuine categories of contemplative practices, but some categories (e.g., walking, exercise, or gardening) may or may not be undertaken in a contemplative way, raising questions about their contributions to the present discussion. There is an ongoing debate in the literature regarding how to define and distinguish contemplative practices^42,63,64^. Moreover, there is considerable overlap between practices, e.g., yoga can include meditation, breathing techniques and relaxation. We cannot discern from our data how participants resolved this overlap, except that they self-identified which practices they engaged in. Asking participants whether and what type of contemplation they practice led to varied responses. In this respect, future studies should favour specific questions and checklists over generic single-item questions and provide clear, distinctive definitions to support participants’ understanding of labels.

In addition, each category represents a wide range of various practices in itself making it hard to reliable deduce which kinds of practice are beneficial^65^. For yoga, different combinations of its four key components (postures, breathing techniques, meditation, ethics) were differentially helpful for specific variables and clinical conditions^12^. Similarly, different meditation techniques had both common and specific effects on participants in comparative studies^66,67^. It is hence imperative to establish which versions of contemplative practices are beneficial for whom to reduce possible adverse effects and increase their effectiveness. Other areas that need to be clarified in both research and practice include questions of intervention delivery, teacher qualification, adaptations to specific populations and individual needs, and the implementation into mental healthcare and referral practices^5,63,68^. Given the widespread use of contemplative practices in general and specific practices like meditation and yoga, more research as well as clinical guidelines are needed. While our data highlights this issue specifically for Australasia, other recent data from our group and others suggests comparable widespread use globally. Stakeholders (such as governmental agencies), intervention providers, practitioners and consumers need to have clarity and transparency regarding what constitutes a safe, useful and evidence-based practice.

## Supplementary Information

Below is the link to the electronic supplementary material.

Supplementary Material 1

## Data Availability

All data, analysis code and supplementary materials are openly available at the Open Science Framework [https://osf.io/4jzwf].

## Declarations

### AI statement

During the preparation of this work the authors used Spark AI, the University of Melbourne’s internal platform, to summarise and rewrite parts of the manuscript. After using this tool, the authors reviewed and edited the content as needed and take full responsibility for the content of the published article.
