# Supplementary material for: Contemplative practices serve as complementary mental health strategies in nationally representative samples from Australia and New Zealand
Source: Sci Rep. 2026 May 19;16:22765. doi: 10.1038/s41598-026-51375-4 (PMC13385573; doi:10.1038/s41598-026-51375-4)
Supplement: Supplementary file 1 — Supplementary Material 1 [file 41598_2026_51375_MOESM1_ESM.docx]

Supplementary Material

Contemplative practices serve as complementary mental health strategies in nationally representative samples from Australia and New Zealand

Karin Matko^1*^, Cate Bailey^1,2^, Julieta Galante^1^, Jonathan N Davies^1^, Nicholas T Van Dam^1^

^1^Contemplative Studies Centre, Melbourne School of Psychological Sciences, University of Melbourne

^2^Melbourne Health Economics, School of Population and Global Health, University of Melbourne

*Corresponding author: karin.matko@unimelb.edu.au

# Section A

## Figure A1. Participants’ main practice and their assigned survey practice


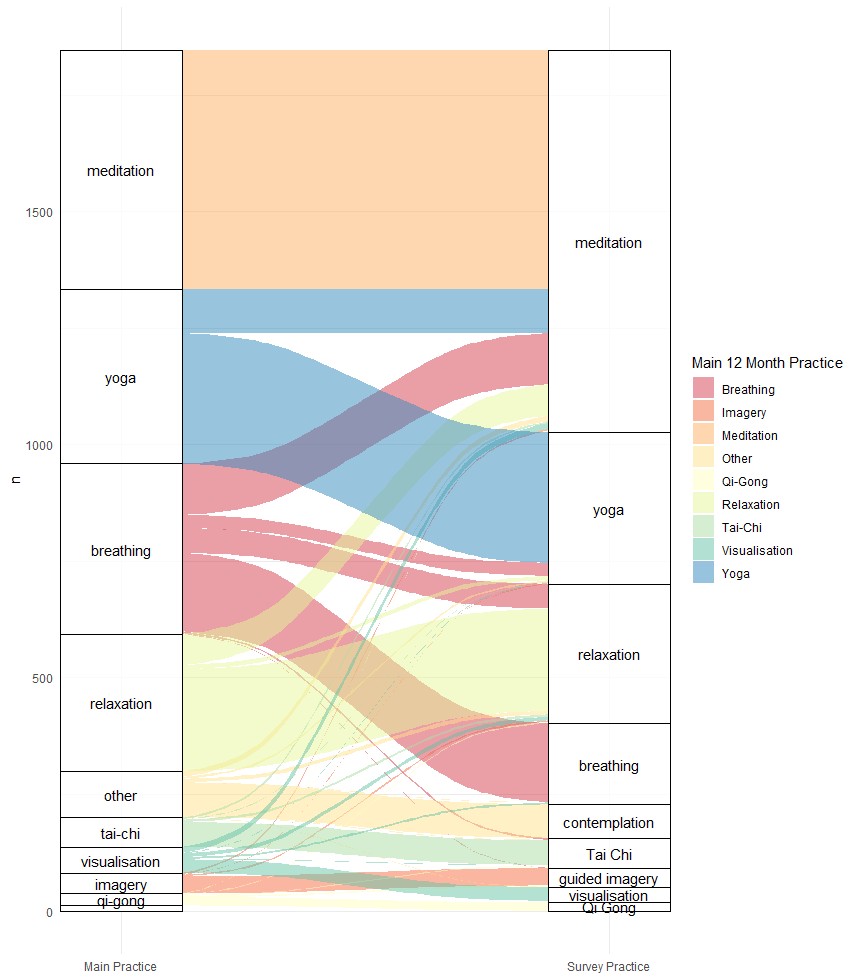


With respect to the four key practices identified earlier, only 63% of participants who responded to further meditation questions, actually reported meditation as their main practice. Likewise, only 75% of yoga practitioners responded to yoga questions, 74% of relaxation practitioners to relaxation questions, and 46% of breathing practitioners to breathing questions.

## Table A2. Variables and their simplification for use in this study

| Gender | Simplified to binary (male/female), “non-binary” or “prefer not so say” coded as NA |
| --- | --- |
| LGBTQIA+ | Binary (yes/no), “prefer not so say” coded as NA |
| Education | Table: Simplified to binary (degree below/ above university)  Regression: Original scale as orthogonal polynomials |
| Income | Recoded response options 1 and 2 in both AU (<$52,000) and NZ (<$30,000) as “low”, options 3 and 4 as “middle” (AU: $52,000−$156,000; NZ: $30,000−$70,000), options 5 and 6 as “high” income (AU: >$156,000; NZ: >$70,000) to merge and simplify country-specific brackets.  Table: Simplified to binary (high/ low or middle income)  Regression: Recoded scale as orthogonal polynomials |
| Occupation | Simplified to binary (working part- or full-time/ not working) |
| Chronic disease | Simplified to binary (yes/no) |
| Disability | Binary (yes/no), “prefer not so say” coded as NA |
| Caring for someone | Simplified to binary (yes/no) |
| Indigenous | Simplified to binary (yes/no) across both countries |
| Religion | Simplified to binary (any religion/ no religion) |
| Spiritual | Table: Simplified to binary (not at all/ more than slightly)  Regression: Original scale as orthogonal polynomials |
| Religious | Table: Simplified to binary (not at all/ more than slightly)  Regression: Original scale as orthogonal polynomials |
| Connected to higher power | Table: Simplified to binary (not at all/ more than slightly) |
|  | Regression: Original scale as orthogonal polynomials |
| Connected to all humanity | Table: Simplified to binary (not at all/ more than slightly)  Regression: Original scale as orthogonal polynomials |
| Connected to nature | Table: Simplified to binary (not at all/ more than slightly)  Regression: Original scale as orthogonal polynomials |
| No alcohol use | Simplified to binary (never/ more than yearly) |
| Non-smoker | Simplified to binary (never/ more than yearly) |
| No cannabis use | Simplified to binary (never/ more than yearly) |
| No psychedelics use | Simplified to binary (never/ more than yearly) |

# Section B – Prevalence and Correlations

## Figure B1. Percentages of meditation, breathing, relaxation, and yoga being chosen as a lifetime, 12-month or main practice


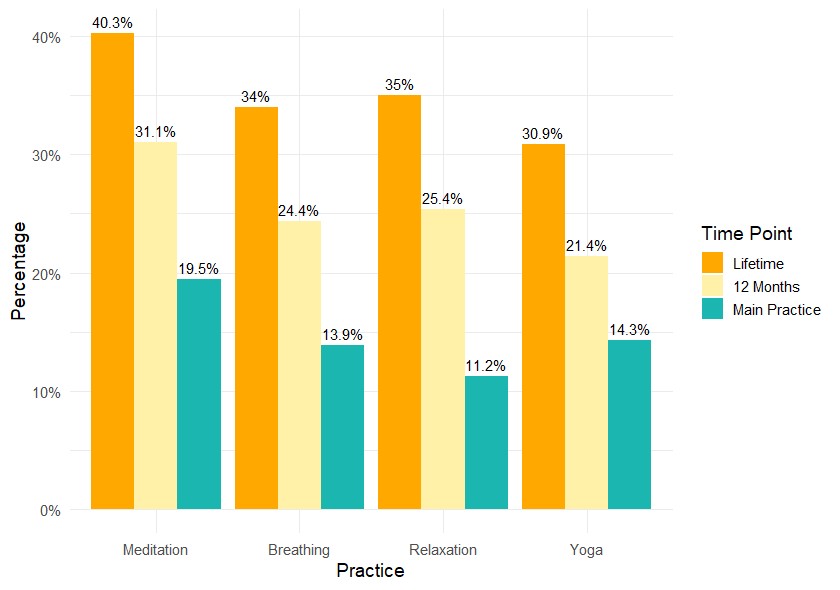


*Note*. Maximum frequency in each category was n = 2640. Selection of multiple responses was possible for lifetime and 12-month practice.

Of all participants who reported lifetime meditation practice, 77.1% reported having practiced meditation in the past 12 months and 48.2% selected it as their main practice. For yoga, 69.2% of lifetime practitioners had practiced in the past 12 months and 46.0% selected it as their main practice. For relaxation and breathing, percentage practice over the past 12 months and main practice were 72.5% and 31.9%, and 71.7% and 40.7% respectively.

## Figure B2. Significant correlations larger than *r* = .20 among lifetime (A) and 12-month (B) contemplative practices


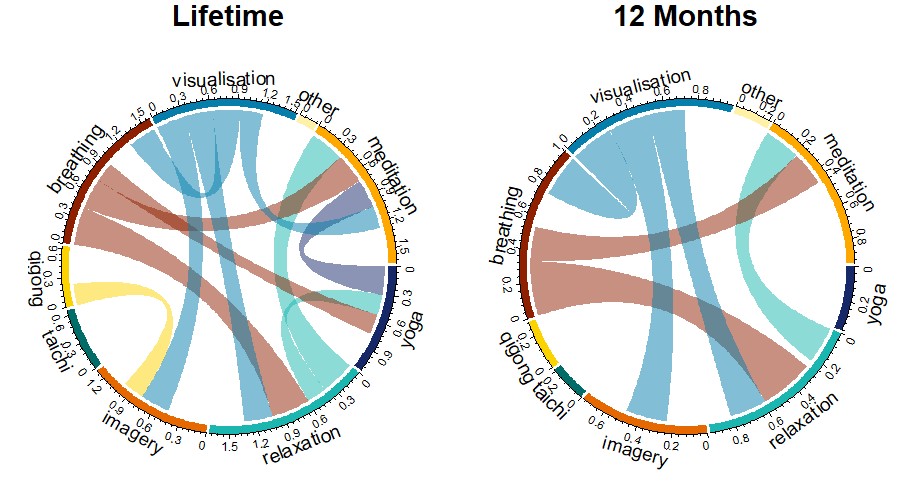


A

B

*Note*. Thickness of lines indicates the strength of the correlation.

Figure B2 shows correlations between all practices that tended to be selected simultaneously and were thus commonly practiced together. Examining only those practices that were correlated across both time windows, we observed the following. Meditation was associated with relaxation and breathing. Yoga showed no robust associations. Relaxation was associated with meditation, breathing, and visualisation. Imagery was associated with visualization. Breathing was associated with meditation and relaxation. Neither Tai Chi nor Qigong exhibited robust associations. Generally, there were fewer correlations at 12 months suggesting participants tended to combine more practices over lifetime than in the past 12 months.

# Section C – Styles of Contemplative Practice

## Table C1. Categories of other contemplative practices, number and total sample proportion of entries per category

|  | n | % of total sample |
| --- | --- | --- |
| Walking | 65 | 3.5 |
| Prayer | 57 | 3.1 |
| Gardening | 42 | 2.3 |
| Sports, exercise | 33 | 1.8 |
| Nature | 14 | 0.8 |
| Music | 13 | 0.7 |
| Reflection | 10 | 0.5 |
| Journaling | 5 | 0.3 |
| Hypnosis | 4 | 0.2 |
| Book reading | 3 | 0.2 |
| Sleep, dream | 3 | 0.2 |

What types of contemplative practice do participants report?

After participants were assigned a survey practice for the contemplative block, they were asked to describe what particular style they practiced. We followed a similar approach in analysing responses to this open-ended question as we did above with their reports on “other contemplative practices”. First, we analyzed all responses by meditators (n=820) and then all responses by participants practicing any other contemplative practice.

## Meditators

We categorized 817 entries into 6 clusters and 46 categories. Table C2 summarises all clusters and categories and the number of entries per cluster/category.

### Table C2. Clusters and categories of practice style and number of entries per category for meditation

| **Cluster** | **Category** | **n** | **n (total)** |
| --- | --- | --- | --- |
| Tradition | yoga | 35 | 56 |
|  | taoist | 6 |  |
|  | buddhist | 5 |  |
|  | zen | 4 |  |
|  | christian | 3 |  |
|  | transcendental | 3 |  |

| Technique | breath | 122 | 348 |
| --- | --- | --- | --- |
|  | mindfulness | 69 |  |
|  | sitting in silence | 51 |  |
|  | walking | 18 |  |
|  | music | 13 |  |
|  | sound | 12 |  |
|  | sensory | 8 |  |
|  | visualisation | 8 |  |
|  | concentration | 7 |  |
|  | mantra | 7 |  |
|  | brahmavihara | 6 |  |
|  | observing | 6 |  |
|  | body scan | 5 |  |
|  | body | 4 |  |
|  | spiritual | 4 |  |
|  | contemplation | 3 |  |
|  | nidra | 3 |  |
|  | dynamic | 2 |  |
| Other contemplative | prayer | 19 | 36 |
|  | sports, exercise | 8 |  |
|  | hypnosis | 5 |  |
|  | nature | 4 |  |
| Aim | relax | 55 | 1 30 |
|  | calm | 19 |  |
|  | clear mind | 11 |  |
|  | comfort | 9 |  |
|  | reflection | 9 |  |
|  | self | 7 |  |
|  | sleep | 7 |  |
|  | deep | 6 |  |
|  | mind-wandering | 3 |  |
|  | healing | 2 |  |
|  | mood | 2 |  |
| Mode | guided | 103 | 109 |
|  | close eyes | 6 |  |
| Unspecified | unspecified | 45 | 80 |
|  | none | 20 |  |
|  | dont know | 10 |  |
|  | own style | 5 |  |
| Other | other | 61 | 61 |

Asking participants to describe their style of practice can lead to quite varied responses. Only 7% of participants name a meditation tradition and 43% describe a specific meditation technique. Note that the top entries for these categories (yoga and breath) might reflect some participants for whom meditation was not their main practice. Of all other participants, 4% name a contemplative practice that is not considered a “classic” meditation technique, 13% describe a mode rather than a specific style of practice (e.g. guided), 16% name an aim or motivation for practicing (e.g. relaxing), 10% state an unspecific style (e.g. meditation), and 7% of entries could not be classified and fall in the “other” category.

In addition, the contemplative block included questions pertaining to the context of participants’ practice and the specific meditation techniques they were using. Both were multiple-choice questions that allowed participants to select all options that applied to them. Tables C3 and C4 provide an overview of the number and percentage of meditators who selected each option.

### Table C3. Number and percentage of meditators who reported practicing different meditation techniques

| **Meditation Technique** | **Meditators**  **(N=820)** |
| --- | --- |
| Meditation with movement (e.g., walking meditation) | 223 (27.2%) |
| Body-centred meditation (e.g., observing body, breath or energy) | 514 (62.7%) |
| Mindful observation (e.g., observing thoughts or emotions) | 500 (61.0%) |
| Contemplation (e.g., contemplating a question) | 210 (25.6%) |
| Visual concentration (e.g., visualisations or concentrating on an object) | 242 (29.5%) |
| Affect-centred meditation (e.g, lovingkindness or compassion) | 131 (16.0%) |
| Mantra meditation (e.g., repeating mantras, meditating with sound) | 121 (14.8%) |
| Something else | 19 (2.3%) |

### Table C3. Number and percentage of meditators who reported practicing in different meditation contexts

| **Meditation Context** | **Meditators**  **(N=820)** |
| --- | --- |
| I work with a teacher or instructor | 77 (9.4%) |
| I listen to audio or video resources | 341 (41.6%) |
| I use a smartphone app | 239 (29.1%) |
| My practice is self-guided | 336 (41.0%) |
| I practice in a group | 111 (13.5%) |
| I practice alone | 397 (48.4%) |
| I don't practice anymore | 24 (2.9%) |
| Something else | 4 (0.5%) |

The most commonly used meditation techniques were body-centered meditation and mindful observation, and the most common practice contexts were practicing alone, self-guided or listening to audio or video resources. Overall, participants practiced in a wide range of contexts and used various meditation techniques.

## Other Contemplative Practices

Then, we analysed the responses of participants engaging in other contemplative practices. We categorized 1018 entries into 18 clusters and 42 categories. As breathing was a large category in this group, we further differentiated different styles of breathing in our coding system. Our coding system was general, i.e., not specific to each practice, which meant that different practices could receive the same code. Unfortunately, participant responses were too short and nonspecific to devise mutually exclusive categories. Hence, when we summarised how often each category appeared in each practice, we did not further distinguish between codes. In addition, when we looked at the number of entries in each practice, we found categories rather than clusters were more helpful in understanding the different styles in each practice. Hence, Table C5 summarises all categories and number of entries per category in each contemplative practice. To ease interpretation, we only report cells with more than 4 entries.

### Table C5. Contemplative practices with categories of practice style and number of entries per category for contemplative practices other than meditation

| **Practice** | **Category** | **n** |
| --- | --- | --- |
| Breathing | breath other | 67 |
|  | breath deep | 53 |
|  | other | 14 |
|  | breath slow | 10 |
|  | breath box | 6 |
|  | calm | 5 |
| Relaxation | breath other | 55 |
|  | other | 40 |
|  | breath deep | 29 |
|  | relax | 21 |
|  | music | 20 |
|  | sitting in silence | 13 |
|  | walking | 12 |
|  | none | 11 |
|  | body | 9 |
|  | mindfulness | 8 |
|  | calm | 7 |
|  | meditation | 7 |
|  | sleep | 7 |
|  | visualisation | 7 |
|  | clear mind | 6 |
|  | yoga yin | 6 |
|  | prayer | 5 |
|  | floating | 4 |
|  | sports, exercise | 4 |
|  | yoga other | 4 |
| Yoga | yoga other | 75 |
|  | other | 33 |
|  | yoga flow | 24 |
|  | yoga hatha | 23 |
|  | yoga bikram | 20 |
|  | sports, exercise | 18 |
|  | don’t know | 17 |
|  | relax | 17 |
|  | stretching | 16 |
|  | breath other | 14 |
|  | unspecified | 12 |
|  | yoga yin | 10 |
|  | body | 7 |
|  | beginner | 6 |
|  | guided/video | 6 |
|  | meditation | 6 |
|  | none | 5 |
| Guided imagery | none | 13 |
|  | other | 8 |
| Visualisation | other | 9 |
|  | visualisation | 9 |
| Tai Chi | other | 11 |
|  | tai chi | 10 |
|  | don't know | 9 |
|  | calm | 7 |
|  | none | 7 |
| Qi Gong | qi gong | 7 |
|  | other | 4 |
| Other contempla-  tive practice | walking prayer | 23  14 |
|  | gardening | 12 |
|  | other | 9 |

Table C5 reveals that similar styles of practice can be found across various contemplative practices. For example, participants described breathing exercises as a style of breathing, relaxation and yoga. Often, participants did not know what their particular style was or wrote “none”. The “other” category reflects styles of practice that could not be categorized as they were either too specific or unspecific. Participants engaging in “other contemplative practices” often described walking, prayer or gardening as their style of practice.

# Section D – Differences in Psychological Distress

## Table D1. Pairwise t-tests for distress scores comparing practitioners of the four main contemplative practices and non-practitioners

| **Group1** None | **Group2**  Meditation | ***M1*** 5.12 | ***M2*** 8.65 | ***t*** | ***df*** | **adjusted *p*** |  | ***d*** |
| --- | --- | --- | --- | --- | --- | --- | --- | --- |
|  |  |  |  | -11.58 | 1158.0 | 0.000 | **** | -0.65 |
| None | Breathing | 5.12 | 8.68 | -9.81 | 696.4 | 0.000 | **** | -0.62 |
| None | Relaxation | 5.12 | 7.32 | -5.57 | 514.3 | 0.000 | **** | -0.38 |
| None | Yoga | 5.12 | 8.14 | -8.62 | 745.2 | 0.000 | **** | -0.54 |
| Meditation | Breathing | 8.65 | 8.68 | -0.06 | 732.1 | 0.949 | ns | 0.00 |
| Meditation | Relaxation | 8.65 | 7.32 | 3.27 | 557.9 | 0.002 | ** | 0.24 |
| Meditation | Yoga | 8.65 | 8.14 | 1.38 | 772.2 | 0.209 | ns | 0.09 |
| Breathing | Relaxation | 8.68 | 7.32 | 3.00 | 626.9 | 0.005 | ** | 0.24 |
| Breathing | Yoga | 8.68 | 8.14 | 1.28 | 735.2 | 0.224 | ns | 0.09 |
| Relaxation | Yoga | 7.32 | 8.14 | -1.87 | 618.2 | 0.089 | ns | -0.15 |

*Note.* Subsample sizes: nnone = 794, nmeditation = 512, nbreathing = 365, nrelaxation = 295, nyoga = 375.

## Table D2. Full covariate model predicting psychological distress prior to reduction

|  | 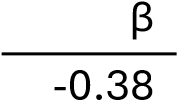 | *p* value | significance |
| --- | --- | --- | --- |
| Age |  | 0.000 | *** |
| Gender (woman) | 0.08 | 0.000 | *** |
| Income | -0.05 | 0.011 | * |
| Education | -0.06 | 0.001 | *** |
| Indigenous | -0.02 | 0.368 | ns |
| Disability | 0.07 | 0.000 | *** |
| Carer | 0.04 | 0.024 | * |
| LGBTQIA+ | 0.06 | 0.001 | *** |
| Working | -0.02 | 0.257 | ns |
| Physical Activity | 0.01 | 0.459 | ns |
| Chronic Disease | 0.12 | 0.000 | *** |
| No Alcohol Use | -0.02 | 0.229 | ns |
| Non-Smoker | -0.06 | 0.004 | ** |
| No Cannabis Use | -0.06 | 0.007 | ** |
| No Psychedelics Use | -0.15 | 0.000 | *** |
| Religion (yes) | -0.05 | 0.022 | * |
| Religiousness | 0.08 | 0.001 | ** |
| Spirituality | 0.02 | 0.368 | ns |
| Connected to Higher Power | 0.06 | 0.021 | * |
| Connected to All Humanity | -0.07 | 0.000 | *** |
| Connected to Nature | -0.03 | 0.131 | ns |

*Note*. *p<0.05; **p<0.01; ***p<0.001

## Table D3. Regression estimates for past year meditation practice predicting psychological distress

0.041

Psychological Distress

(1)

(2)

(3)

(4)

Meditation 12

-

month

0.164

***

**

0.008

0.031

-

0.314

***

-

0.310

***

Age -0.363^***^

| Gender (woman) |  | 0.078^***^ | 0.077^***^ | 0.077^***^ |
| --- | --- | --- | --- | --- |
| Income |  | -0.053^***^ | -0.051^***^ | -0.050^***^ |
| Education |  | -0.067^***^ | -0.078^***^ | -0.078^***^ |
| Disability |  | 0.070^***^ | 0.053^***^ | 0.054^***^ |
| Carer |  | 0.037^**^ | 0.023 | 0.024 |
| LGBTQIA+ |  | 0.054^***^ | 0.036^**^ | 0.040^**^ |
| Chronic Disease |  | 0.118^***^ | 0.095^***^ | 0.095^***^ |
| Non-Smoker |  | -0.054^***^ | -0.025 | -0.025 |
| No Cannabis Use |  | -0.062^***^ | -0.056^**^ | -0.058^**^ |
| No Psychedelics Use |  | -0.150^***^ | -0.132^***^ | -0.133^***^ |
| Religion (yes) |  | -0.045^**^ | -0.043^**^ | -0.044^**^ |
| Religiousness |  | 0.083^***^ | 0.089^***^ | 0.091^***^ |
| Connected to Higher Power |  | 0.055^**^ | 0.037^*^ | 0.034 |
| Connected to All Humanity |  | -0.086^***^ | -0.072^***^ | -0.071^***^ |
| Mental HC (unmet) |  |  | 0.163^***^ | 0.209^***^ |
| Mental HC (yes) |  |  | 0.192^***^ | 0.194^***^ |
| Meditation * Mental HC (unmet) |  |  |  | -0.073^***^ |
| Meditation * Mental HC (yes) |  |  |  | -0.011 |
| Observations | 2,640 | 2,598 | 2,598 | 2,598 |
| R^2^ | 0.027 | 0.329 | 0.37 | 0.372 |
| Adjusted R^2^ | 0.026 | 0.325 | 0.365 | 0.367 |

*Note*. HC = healthcare; *p<0.05; **p<0.01; ***p<0.001

## D4 relaxation practice in the past 12 months predicting psychological distress across 4 models

Psychological Distress

(1)

(2)

(3)

(4)

| Relaxation 12-month | 0.075^***^ | 0.019 |
| --- | --- | --- |
| Age |  | -0.368^***^ |
| Gender (woman) |  | 0.078^***^ |
| Income |  | -0.054^**^ |
| Education |  | -0.063^***^ |
| Disability |  | 0.069^***^ |
| Carer |  | 0.036^*^ |
| LGBTQIA+ |  | 0.057^**^ |
| Chronic Disease |  | 0.118^***^ |
| Non-Smoker |  | -0.055^**^ |
| No Cannabis Use |  | -0.062^**^ |
| No Psychedelics Use |  | -0.151^***^ |
| Religion (yes) |  | -0.047^*^ |
| Religiousness |  | 0.083^***^ |
| Connected to Higher Power |  | 0.060^**^ |
| Connected to All Humanity |  | -0.084^***^ |


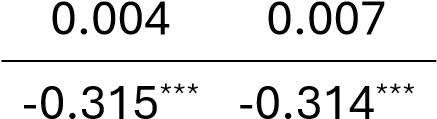
 0.077^***^ 0.077^***^

-0.051^**^ -0.050^**^

-0.077^***^ -0.077^***^

0.053^**^ 0.052^**^

0.023 0.023

0.036^*^ 0.037^*^

0.095^***^ 0.096^***^

-0.025 -0.026

-0.056^*^ -0.055^*^

-0.132^***^ -0.133^***^

-0.043^*^ -0.044^*^

0.089^***^ 0.092^***^

0.038 0.037

-0.071^***^ -0.072^***^

Mental HC (unmet)
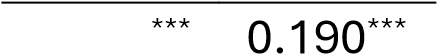
0.163

Mental HC (yes) 0.193^***^ 0.181^***^

Relaxation * Mental HC (unmet)

Relaxation * Mental HC (yes)

-

0.049

*

0.021

2,598

Observations 2,640 2,598 2,598

R^2^ 0.006 0.328 0.37 0.372

Adjusted R^2^ 0.005 0.324 0.365 0.367

*Note*. HC = healthcare; *p<0.05; **p<0.01; ***p<0.001

## D5 breathing practice in the past 12 months predicting psychological distress across 4 models

(2)

Psychological Distress

(1)

(3)

(4)

Breathing 12

-

month

0.110

***

***

0.051

**

0.042

*

-

0.310

***

-

0.310

***

0.073

Age -0.359^***^

| Gender (woman) |  | 0.072^***^ | | 0.072^***^ | | 0.072^***^ | |
| --- | --- | --- | --- | --- | --- | --- | --- |
| Income |  | -0.054^**^ | | -0.051^**^ | | -0.051^**^ | |
| Education |  | -0.064^***^ | | -0.077^***^ | | -0.078^***^ | |
| Disability |  | 0.064^***^ | | 0.049^**^ | | 0.049^**^ | |
| Carer |  | 0.040^*^ | | 0.025 | | 0.026 | |
| LGBTQIA+ |  | 0.060^***^ | | 0.039^*^ | | 0.039^*^ | |
| Chronic Disease |  | 0.116^***^ | | 0.094^***^ | | 0.093^***^ | |
| Non-Smoker |  | -0.055^**^ | | -0.025 | | -0.026 | |
| No Cannabis Use |  | -0.059^*^ | | -0.053^*^ | | -0.053^*^ | |
| No Psychedelics Use |  | -0.160^***^ | | -0.140^***^ | | -0.140^***^ | |
| Religion (yes) |  | -0.046^*^ | | -0.042^*^ | | -0.042^*^ | |
| Religiousness |  | 0.089^***^ | | 0.094^***^ | | 0.095^***^ | |
| Connected to Higher Power |  | 0.050^*^ | | 0.03 | | 0.029 | |
| Connected to All Humanity |  | -0.087^***^ | | -0.074^***^ | | -0.074^***^ | |
| Mental HC (unmet) |  |  | | 0.161^***^ | | 0.164^***^ | |
| Mental HC (yes) | |  |  | | 0.188^***^ | | 0.176^***^ |
| Breathing * Mental HC (unmet) | |  |  | |  | | -0.006 |
| Breathing * Mental HC (yes) | |  |  | |  | | 0.024 |
| Observations | | 2,640 | 2,598 | | 2,598 | | 2,598 |
| R^2^ | | 0.012 | 0.332 | | 0.372 | | 0.372 |
| Adjusted R^2^ | | 0.012 | 0.328 | | 0.368 | | 0.368 |

*Note*. HC = healthcare; *p<0.05; **p<0.01; ***p<0.001

## D6 yoga practice in the past 12 months predicting psychological distress across 4 models

Psychological Distress

(1)

(2)

(3)

(4)

| Yoga 12-month | 0.092^***^ | -0.026 |
| --- | --- | --- |
| Age |  | -0.373^***^ |
| Gender (woman) |  | 0.084^***^ |
| Income |  | -0.053^**^ |
| Education |  | -0.058^***^ |
| Disability |  | 0.068^***^ |
| Carer |  | 0.038^*^ |
| LGBTQIA+ |  | 0.057^**^ |
| Chronic Disease |  | 0.120^***^ |
| Non-Smoker |  | -0.055^**^ |
| No Cannabis Use |  | -0.065^**^ |
| No Psychedelics Use |  | -0.149^***^ |
| Religion (yes) |  | -0.047^*^ |
| Religiousness |  | 0.080^***^ |
| Connected to Higher Power |  | 0.064^**^ |
| Connected to All Humanity |  | -0.081^***^ |


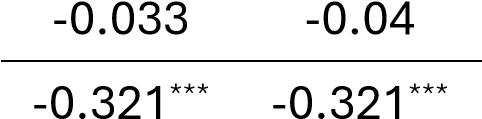
 0.082^***^ 0.083^***^

-0.050^**^ -0.050^**^

-0.072^***^ -0.072^***^

0.051^**^ 0.052^**^

0.024 0.024

0.037^*^ 0.036^*^

0.096^***^ 0.095^***^

-0.023 -0.024

-0.058^**^ -0.058^**^

-0.131^***^ -0.131^***^

-0.043^*^ -0.043^*^

0.087^***^ 0.088^***^

0.04 0.04

-0.068^***^ -0.069^***^

Mental HC (unmet)
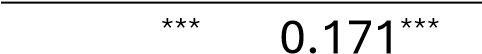
0.165

| Mental HC (yes) |  |  | 0.194^***^ | 0.185^***^ |
| --- | --- | --- | --- | --- |
| Yoga * Mental HC (unmet) |  |  |  | -0.011 |
| Yoga * Mental HC (yes) |  |  |  | 0.021 |
| Observations | 2,640 | 2,598 | 2,598 | 2,598 |
| R^2^ | 0.008 | 0.328 | 0.371 | 0.371 |
| Adjusted R^2^ | 0.008 | 0.324 | 0.366 | 0.366 |

*Note*. HC = healthcare; *p<0.05; **p<0.01; ***p<0.001

# Section E – Full demographic descriptives by subsample

**None Meditation Breathing Relaxation Yoga**

**(N=794) (N=512) (N=365) (N=295) (N=375)**

| **Age group** |  |  |  |  |  |
| --- | --- | --- | --- | --- | --- |
| 18-29 | 91 (11.5%) | 124 (24.2%) | 112 (30.7%) | 46 (15.6%) | 126 (33.6%) |
| 30-39 | 97 (12.2%) | 148 (28.9%) | 67 (18.4%) | 50 (16.9%) | 98 (26.1%) |
| 40-49 | 94 (11.8%) | 96 (18.8%) | 57 (15.6%) | 60 (20.3%) | 72 (19.2%) |
| 50-59 | 152 (19.1%) | 76 (14.8%) | 42 (11.5%) | 51 (17.3%) | 39 (10.4%) |
| 60+ | 360 (45.3%) | 68 (13.3%) | 87 (23.8%) | 88 (29.8%) | 40 (10.7%) |
| **Gender** |  |  |  |  |  |
| Man | 462 (58.2%) | 259 (50.6%) | 137 (37.5%) | 122 (41.4%) | 128 (34.1%) |
| Woman | 330 (41.6%) | 252 (49.2%) | 225 (61.6%) | 173 (58.6%) | 246 (65.6%) |
| Non-binary | 2 (0.3%) | 1 (0.2%) | 3 (0.8%) | 0 (0%) | 1 (0.3%) |
| **LGBTQIA+ status** |  |  |  |  |  |
| No | 757 (95.3%) | 380 (74.2%) | 314 (86.0%) | 258 (87.5%) | 291 (77.6%) |
| Yes | 32 (4.0%) | 125 (24.4%) | 46 (12.6%) | 37 (12.5%) | 83 (22.1%) |
| **Education** |  |  |  |  |  |
| Less than Primary | 1 (0.1%) | 2 (0.4%) | 0 (0%) | 0 (0%) | 0 (0%) |
| Primary | 5 (0.6%) | 0 (0%) | 1 (0.3%) | 1 (0.3%) | 4 (1.1%) |
| Some Secondary | 114 (14.4%) | 16 (3.1%) | 27 (7.4%) | 24 (8.1%) | 12 (3.2%) |
| Secondary | 179 (22.5%) | 78 (15.2%) | 73 (20.0%) | 48 (16.3%) | 45 (12.0%) |
| Vocational or Similar | 209 (26.3%) | 86 (16.8%) | 82 (22.5%) | 71 (24.1%) | 59 (15.7%) |
| Some University but no degree | 63 (7.9%) | 70 (13.7%) | 37 (10.1%) | 36 (12.2%) | 58 (15.5%) |
| University - Bachelors Degree  Graduate or professional degree (MA, | 173 (21.8%) | 201 (39.3%) | 109 (29.9%) | 84 (28.5%) | 141 (37.6%) |
| MS, MBA, PhD, Law Degree, Medical Degree etc) | 50 (6.3%) | 59 (11.5%) | 36 (9.9%) | 31 (10.5%) | 56 (14.9%) |
| **Employment status** |  |  |  |  |  |
| Working full-time | 292 (36.8%) | 322 (62.9%) | 146 (40.0%) | 138 (46.8%) | 264 (70.4%) |
| Working part-time | 134 (16.9%) | 89 (17.4%) | 91 (24.9%) | 47 (15.9%) | 50 (13.3%) |

| Studying | 13 (1.6%) | 10 (2.0%) | 23 (6.3%) | 7 (2.4%) | 11 (2.9%) |
| --- | --- | --- | --- | --- | --- |
| Unemployed and looking for work | 43 (5.4%) | 18 (3.5%) | 18 (4.9%) | 16 (5.4%) | 12 (3.2%) |
| Unemployed and not looking for work | 35 (4.4%) | 12 (2.3%) | 20 (5.5%) | 12 (4.1%) | 7 (1.9%) |
| Retired | 250 (31.5%) | 48 (9.4%) | 58 (15.9%) | 66 (22.4%) | 26 (6.9%) |
| Other | 27 (3.4%) | 13 (2.5%) | 9 (2.5%) | 9 (3.1%) | 5 (1.3%) |
| **Chronic health condition** |  |  |  |  |  |
| No | 437 (55.0%) | 264 (51.6%) | 173 (47.4%) | 142 (48.1%) | 208 (55.5%) |
| Yes | 357 (45.0%) | 248 (48.4%) | 192 (52.6%) | 153 (51.9%) | 167 (44.5%) |
| **Disability** |  |  |  |  |  |
| No | 682 (85.9%) | 465 (90.8%) | 296 (81.1%) | 246 (83.4%) | 347 (92.5%) |
| Yes | 108 (13.6%) | 44 (8.6%) | 65 (17.8%) | 47 (15.9%) | 26 (6.9%) |
| Missing | 4 (0.5%) | 3 (0.6%) | 4 (1.1%) | 2 (0.7%) | 2 (0.5%) |
| **Carer responsibilities** |  |  |  |  |  |
| No | 577 (72.7%) | 244 (47.7%) | 225 (61.6%) | 157 (53.2%) | 168 (44.8%) |
| Yes | 217 (27.3%) | 268 (52.3%) | 140 (38.4%) | 138 (46.8%) | 207 (55.2%) |
| **Indigenous status** |  |  |  |  |  |
| No | 730 (91.9%) | 389 (76.0%) | 321 (87.9%) | 224 (75.9%) | 253 (67.5%) |
| Yes | 64 (8.1%) | 123 (24.0%) | 44 (12.1%) | 71 (24.1%) | 122 (32.5%) |
| **Religion (current)** |  |  |  |  |  |
| No religion  Christian (including Church of England, | 417 (52.5%) | 238 (46.5%) | 187 (51.2%) | 121 (41.0%) | 174 (46.4%) |
| Catholic, Protestant and all other Christian denominations) | 322 (40.6%) | 179 (35.0%) | 139 (38.1%) | 125 (42.4%) | 115 (30.7%) |
| Hindu | 3 (0.4%) | 29 (5.7%) | 4 (1.1%) | 6 (2.0%) | 23 (6.1%) |
| Jewish | 4 (0.5%) | 10 (2.0%) | 2 (0.5%) | 5 (1.7%) | 10 (2.7%) |
| Muslim | 16 (2.0%) | 18 (3.5%) | 3 (0.8%) | 6 (2.0%) | 17 (4.5%) |
| Buddhist | 7 (0.9%) | 18 (3.5%) | 9 (2.5%) | 12 (4.1%) | 9 (2.4%) |
| Sikh  Indigenous religions / spiritual prac- | 1 (0.1%) | 5 (1.0%) | 2 (0.5%) | 3 (1.0%) | 5 (1.3%) |
| tices (e.g., Australian Aboriginal, Māori spirituality, Polynesian spirituality, Hawaiian Religion, etc) | 7 (0.9%) | 5 (1.0%) | 6 (1.6%) | 8 (2.7%) | 13 (3.5%) |

| Other religion | 17 (2.1%) | 10 (2.0%) | 13 (3.6%) | 9 (3.1%) | 9 (2.4%) |
| --- | --- | --- | --- | --- | --- |
| **Spiritual** |  |  |  |  |  |
| Not at all | 374 (47.1%) | 50 (9.8%) | 61 (16.7%) | 51 (17.3%) | 71 (18.9%) |
| Slightly | 226 (28.5%) | 161 (31.4%) | 149 (40.8%) | 102 (34.6%) | 120 (32.0%) |
| Moderately | 107 (13.5%) | 215 (42.0%) | 109 (29.9%) | 85 (28.8%) | 132 (35.2%) |
| Very | 87 (11.0%) | 86 (16.8%) | 46 (12.6%) | 57 (19.3%) | 52 (13.9%) |
| **Religious** |  |  |  |  |  |
| Not at all | 451 (56.8%) | 146 (28.5%) | 167 (45.8%) | 108 (36.6%) | 141 (37.6%) |
| Slightly | 178 (22.4%) | 155 (30.3%) | 104 (28.5%) | 87 (29.5%) | 105 (28.0%) |
| Moderately | 104 (13.1%) | 153 (29.9%) | 64 (17.5%) | 66 (22.4%) | 100 (26.7%) |
| Very | 61 (7.7%) | 58 (11.3%) | 30 (8.2%) | 34 (11.5%) | 29 (7.7%) |
| **Connected to higher power** |  |  |  |  |  |
| Not at all | 433 (54.5%) | 71 (13.9%) | 119 (32.6%) | 77 (26.1%) | 97 (25.9%) |
| Slightly | 177 (22.3%) | 146 (28.5%) | 116 (31.8%) | 82 (27.8%) | 106 (28.3%) |
| Moderately | 96 (12.1%) | 198 (38.7%) | 83 (22.7%) | 88 (29.8%) | 114 (30.4%) |
| Very | 88 (11.1%) | 97 (18.9%) | 47 (12.9%) | 48 (16.3%) | 58 (15.5%) |
| **Connection to nature** |  |  |  |  |  |
| Not at all | 141 (17.8%) | 24 (4.7%) | 23 (6.3%) | 25 (8.5%) | 21 (5.6%) |
| Slightly | 309 (38.9%) | 158 (30.9%) | 116 (31.8%) | 89 (30.2%) | 123 (32.8%) |
| Moderately | 242 (30.5%) | 208 (40.6%) | 142 (38.9%) | 116 (39.3%) | 146 (38.9%) |
| Very | 102 (12.8%) | 122 (23.8%) | 84 (23.0%) | 65 (22.0%) | 85 (22.7%) |
| **Connection to all humanity** |  |  |  |  |  |
| Not at all | 196 (24.7%) | 41 (8.0%) | 61 (16.7%) | 36 (12.2%) | 42 (11.2%) |
| Slightly | 314 (39.5%) | 185 (36.1%) | 149 (40.8%) | 102 (34.6%) | 138 (36.8%) |
| Moderately | 217 (27.3%) | 213 (41.6%) | 117 (32.1%) | 108 (36.6%) | 140 (37.3%) |
| Very | 67 (8.4%) | 73 (14.3%) | 38 (10.4%) | 49 (16.6%) | 55 (14.7%) |
| **Alcohol use (past year)** |  |  |  |  |  |
| Never | 166 (20.9%) | 62 (12.1%) | 53 (14.5%) | 36 (12.2%) | 33 (8.8%) |
| Yearly | 76 (9.6%) | 52 (10.2%) | 43 (11.8%) | 33 (11.2%) | 40 (10.7%) |
| Monthly | 177 (22.3%) | 174 (34.0%) | 110 (30.1%) | 99 (33.6%) | 116 (30.9%) |
| weekly | 259 (32.6%) | 194 (37.9%) | 128 (35.1%) | 98 (33.2%) | 158 (42.1%) |
| Daily | 116 (14.6%) | 30 (5.9%) | 31 (8.5%) | 29 (9.8%) | 28 (7.5%) |

| **Tobacco use (past year)** |  |  |  |  |  |
| --- | --- | --- | --- | --- | --- |
| Never | 588 (74.1%) | 227 (44.3%) | 214 (58.6%) | 170 (57.6%) | 194 (51.7%) |
| Yearly | 11 (1.4%) | 44 (8.6%) | 19 (5.2%) | 20 (6.8%) | 22 (5.9%) |
| Monthly | 23 (2.9%) | 71 (13.9%) | 20 (5.5%) | 25 (8.5%) | 41 (10.9%) |
| weekly | 32 (4.0%) | 87 (17.0%) | 25 (6.8%) | 27 (9.2%) | 56 (14.9%) |
| Daily | 140 (17.6%) | 83 (16.2%) | 87 (23.8%) | 53 (18.0%) | 62 (16.5%) |
| **Cannabis use (past year)** |  |  |  |  |  |
| Never | 719 (90.6%) | 320 (62.5%) | 282 (77.3%) | 235 (79.7%) | 253 (67.5%) |
| Yearly | 25 (3.1%) | 52 (10.2%) | 28 (7.7%) | 17 (5.8%) | 43 (11.5%) |
| Monthly | 18 (2.3%) | 69 (13.5%) | 14 (3.8%) | 17 (5.8%) | 43 (11.5%) |
| weekly | 14 (1.8%) | 52 (10.2%) | 14 (3.8%) | 9 (3.1%) | 19 (5.1%) |
| Daily | 18 (2.3%) | 19 (3.7%) | 27 (7.4%) | 17 (5.8%) | 17 (4.5%) |
| **Psychedelic use (past year)** |  |  |  |  |  |
| Never | 748 (94.2%) | 369 (72.1%) | 337 (92.3%) | 260 (88.1%) | 291 (77.6%) |
| Yearly | 23 (2.9%) | 49 (9.6%) | 25 (6.8%) | 14 (4.7%) | 39 (10.4%) |
| Monthly | 14 (1.8%) | 57 (11.1%) | 3 (0.8%) | 11 (3.7%) | 22 (5.9%) |
| weekly | 8 (1.0%) | 34 (6.6%) | 0 (0%) | 9 (3.1%) | 20 (5.3%) |
| Daily | 1 (0.1%) | 3 (0.6%) | 0 (0%) | 1 (0.3%) | 3 (0.8%) |
|  |  |  |  |  |  |
| **Household income (Australia)** |  |  |  |  |  |
| $0 - $26,000 | 64 (10.9%) | 23 (5.2%) | 26 (10.3%) | 16 (6.9%) | 14 (4.4%) |
| $26,000 - $52,000 | 140 (23.9%) | 47 (10.6%) | 45 (17.9%) | 54 (23.4%) | 39 (12.3%) |
| $52,000 - $91,000 | 186 (31.8%) | 121 (27.2%) | 64 (25.4%) | 57 (24.7%) | 73 (23.1%) |
| $91,000 - $156,000 | 128 (21.9%) | 140 (31.5%) | 71 (28.2%) | 53 (22.9%) | 99 (31.3%) |
| $156,000 - $208,000 | 47 (8.0%) | 77 (17.3%) | 32 (12.7%) | 39 (16.9%) | 62 (19.6%) |
| $208,000+ | 20 (3.4%) | 37 (8.3%) | 14 (5.6%) | 12 (5.2%) | 29 (9.2%) |
| **Ethnicity (Australia)** |  |  |  |  |  |
| Australian | 206 (35.2%) | 98 (22.0%) | 73 (29.0%) | 62 (26.8%) | 84 (26.6%) |
| Australian Aboriginal or Torres Strait Islander | 22 (3.8%) | 14 (3.1%) | 5 (2.0%) | 7 (3.0%) | 13 (4.1%) |
| European (e.g., English, Irish, Scottish, Italian, German) | 266 (45.5%) | 230 (51.7%) | 129 (51.2%) | 109 (47.2%) | 126 (39.9%) |
| Asian (e.g., Chinese, Indian, Filipino) | 54 (9.2%) | 67 (15.1%) | 31 (12.3%) | 23 (10.0%) | 57 (18.0%) |
| Other | 37 (6.3%) | 36 (8.1%) | 14 (5.6%) | 30 (13.0%) | 36 (11.4%) |
| **Remoteness (Australia)** |  |  |  |  |  |
| Major Cities of Australia | 428 (73.2%) | 376 (84.5%) | 200 (79.4%) | 173 (74.9%) | 271 (85.8%) |
| Inner Regional Australia | 109 (18.6%) | 55 (12.4%) | 32 (12.7%) | 41 (17.7%) | 31 (9.8%) |
| Outer Regional Australia | 41 (7.0%) | 12 (2.7%) | 19 (7.5%) | 16 (6.9%) | 13 (4.1%) |
| Remote Australia | 6 (1.0%) | 2 (0.4%) | 1 (0.4%) | 0 (0%) | 1 (0.3%) |
| Very Remote Australia | 1 (0.2%) | 0 (0%) | 0 (0%) | 1 (0.4%) | 0 (0%) |
|  |  |  |  |  |  |
| **Household income (New Zealand)** |  |  |  |  |  |
| $0 - $20,000 | 7 (3.3%) | 1 (1.5%) | 10 (8.8%) | 4 (6.3%) | 2 (3.4%) |
| $20,000 - $30,000 | 22 (10.5%) | 10 (14.9%) | 13 (11.5%) | 4 (6.3%) | 0 (0%) |
| $30,000 - $50,000 | 36 (17.2%) | 10 (14.9%) | 20 (17.7%) | 12 (18.8%) | 1 (1.7%) |
| $50,000 - $70,000 | 36 (17.2%) | 4 (6.0%) | 14 (12.4%) | 13 (20.3%) | 7 (11.9%) |
| $70,000 - $100,000 | 35 (16.7%) | 13 (19.4%) | 20 (17.7%) | 8 (12.5%) | 14 (23.7%) |
| $100,000 - $150,000 | 44 (21.1%) | 15 (22.4%) | 21 (18.6%) | 9 (14.1%) | 19 (32.2%) |
| $150,000+ | 29 (13.9%) | 14 (20.9%) | 15 (13.3%) | 14 (21.9%) | 16 (27.1%) |
| **Ethnicity (New Zealand)** |  |  |  |  |  |
| European | 144 (68.9%) | 36 (53.7%) | 76 (67.3%) | 32 (50.0%) | 30 (50.8%) |
| Māori | 24 (11.5%) | 15 (22.4%) | 17 (15.0%) | 18 (28.1%) | 8 (13.6%) |
| Pacific peoples (e.g., Samoan, Cook Islands Māori, Tongan, Niuean) | 12 (5.7%) | 4 (6.0%) | 7 (6.2%) | 3 (4.7%) | 9 (15.3%) |
| Asian (e.g., Chinese, Indian) | 22 (10.5%) | 11 (16.4%) | 12 (10.6%) | 8 (12.5%) | 12 (20.3%) |
| Middle Eastern / Latin American / African | 5 (2.4%) | 1 (1.5%) | 0 (0%) | 0 (0%) | 0 (0%) |
| Other | 2 (1.0%) | 0 (0%) | 1 (0.9%) | 3 (4.7%) | 0 (0%) |
